# Calf Diarrhea Caused by Prolonged Expansion of Autochthonous Gut *Enterobacteriaceae* and Their Lytic Bacteriophages

**DOI:** 10.1128/mSystems.00816-20

**Published:** 2021-03-02

**Authors:** Tae Woong Whon, Hyun Sik Kim, Na-Ri Shin, Hojun Sung, Min-Soo Kim, Joon Yong Kim, Woorim Kang, Pil Soo Kim, Dong-Wook Hyun, Hoon Je Seong, Woo Jun Sul, Seong Woon Roh, Jin-Woo Bae

**Affiliations:** a Department of Life and Nanopharmaceutical Sciences and Department of Biology, Kyung Hee University, Seoul, Republic of Korea; b Microbiology and Functionality Research Group, World Institute of Kimchi, Gwangju, Republic of Korea; c Biological Resource Center, Korea Research Institute of Bioscience and Biotechnology, Jeongeup-si, Jeollabuk-do, Republic of Korea; d Department of Microbiology and Molecular Biology, Chungnam National University, Daejeon, Republic of Korea; e Department of Systems Biotechnology, Chung-Ang University, Anseong-si, Gyeonggi-do, Republic of Korea; University of Connecticut

**Keywords:** calf diarrhea, gut microbiome, dysbiosis, *Enterobacteriaceae*, bacteriophages

## Abstract

Neonatal calf diarrhea is a common disease leading to a major economic loss for cattle producers worldwide. Several infectious and noninfectious factors are implicated in calf diarrhea, but disease control remains problematic because of the multifactorial etiology of the disease. Here, we conducted diagnostic multiplex PCR assay and meta-omics analysis (16S rRNA gene-based metataxonomics and untargeted transcriptional profiling) of rectal content of normal and diarrheic beef calves (*n *= 111). In the diarrheic calf gut, we detected both microbial compositional dysbiosis (i.e., increased abundances of the family *Enterobacteriaceae* members and their lytic bacteriophages) and functional dysbiosis (i.e., elevated levels of aerobic respiration and virulence potential). The calf diarrheic transcriptome mirrored the gene expression of the bovine host and was enriched in cellular pathways of sulfur metabolism, innate immunity, and gut motility. We then isolated 12 nontoxigenic *Enterobacteriaceae* strains from the gut of diarrheic calves. Feeding a strain mixture to preweaning mice resulted in a significantly higher level of fecal moisture content, with decreased body weight gain and shortened colon length. The presented findings suggest that gut inflammation followed by a prolonged expansion of nontoxigenic autochthonous *Enterobacteriaceae* contributes to the onset of diarrhea in preweaning animals.

**IMPORTANCE** Calf diarrhea is the leading cause of death of neonatal calves worldwide. Several infectious and noninfectious factors are implicated in calf diarrhea, but disease control remains problematic because of the multifactorial etiology of the disease. The major finding of the current study centers around the observation of microbial compositional and functional dysbiosis in rectal samples from diarrheic calves. These results highlight the notion that gut inflammation followed by a prolonged expansion of autochthonous *Enterobacteriaceae* contributes to the onset of calf diarrhea. Moreover, this condition possibly potentiates the risk of invasion of notorious enteric pathogens, including *Salmonella* spp., and the emergence of inflammation-resistant (or antibiotic-resistant) microbiota via active horizontal gene transfer mediated by lytic bacteriophages.

## INTRODUCTION

In the cattle industry worldwide, calf diarrhea is the primary leading cause of death of neonatal calves and is responsible for a major economic loss for cattle producers (1). Surprisingly, the National Animal Health Monitoring System for the U.S. Dairy reported in 2012 that only 5.7% of preweaning heifers were diarrhea free, while 85.7% of calves were undergoing antibiotic treatment because of diarrhea at the time of analysis (2). Because the gastrointestinal tract is a major portal of entry for many biological and/or xenobiotic entities, studies in the last several decades have focused on revealing the causative agents of calf diarrhea by detecting specific pathogens in fecal specimens. Accordingly, several viruses (e.g., the bovine viral diarrhea virus, bovine coronavirus, and group A rotavirus), bacteria (e.g., *Salmonella* spp. and Clostridium perfringens), and protozoa (e.g., Eimeria zuernii) have been listed as the infectious pathogens of calf diarrhea (3, 4). Most recently, however, advanced diagnostic tools (i.e., metagenomics and multiplex real-time PCR panels) were employed for the determination of the microbiological etiology of diarrhea. The approach revealed a high incidence of coinfections in the feces of clinically healthy calves (5).

Besides the above allochthonous etiological agents derived from external environments, which transiently interact with the gut epithelium, a gut-dwelling autochthonous microbiota is also capable of triggering and/or initiating the calf diarrhea. In mammalian neonates, microbial colonization by the major gut microbiota begins after birth (6). Initially, early microbial colonizers from maternal sources (e.g., facultative anaerobes, mainly *Proteobacteria* species) consume the intestinal oxygen and facilitate colonization of subsequent colonizers, such as strict anaerobes (7–9). Importantly, disturbance of this colonization pattern (i.e., the duration of the early colonizer bloom) is linked to an increased risk of neonatal gut diseases (10). Gut inflammation followed by an abnormal composition of gut microbiota (i.e., dysbiosis) increases the frequency of diarrhea (11). Moreover, the successive microbial colonization results in a dense microbial population of the autochthonous bacteria, with a stable population structure, in the gut, conferring colonization resistance against allochthonous pathogens (12, 13). From this perspective, a complicated association between noninfectious factors (e.g., diet types, environmental stresses, and different peripartum calving managements) and the use of antibiotics lead to an incomplete establishment of gut microbiota and further dysbiosis-induced diarrhea in calves.

Microbial commensalism and/or pathogenesis in the mammalian gut are not solely restricted to bacteria, but also involve viral and fungal species. Enteric viruses are central members of the autochthonous microbiota, and most of them are bacterial viruses (bacteriophages) (14). Indeed, several studies have highlighted the associations between compositional alterations in the gut bacteriophage population and microbiome-related diseases (15–17). In a recent study, we demonstrated an intriguing predominance of temperate bacteriophages that lysogenize their host bacteria in the gut environment (18). Only in specific circumstances, e.g., diarrhea, inflammatory signals boost the production of free phages and a subsequent lysogenic conversion of a temperate bacteriophage that infects Salmonella enterica serovar Typhimurium (19). However, a factor(s) triggering global induction of the lytic cycle of the gut prophages and its consequent effects on the progression of diarrhea remain to be identified, especially in economically important animals.

In the current study, we employed metataxonomics (i.e., amplification and sequencing of bacterial 16S rRNA genes) combined with rectal transcriptomics analysis to understand the multifactorial nature of calf diarrhea. We aimed to delineate a detailed tripartite relationship between gut bacteria, the bovine host, and viruses upon diarrheic progression in Korean brown cattle calves (Bos taurus *coreanae*; here referred to as the Hanwoo). We further evaluated the causative role of the alteration of the gut microbiota in the diarrheic symptoms in preweaning mice. Our findings indicate that the increased abundance and/or prolonged expansion of the nontoxigenic *Enterobacteriaceae* in the gut of preweaning animals render the host gut diarrheagenic, and potentiate the risk of pathogen infections.

## RESULTS

### Diagnostic multiplex PCR allows only a partial determination of the etiology of calf diarrhea.

**FIG 1:**
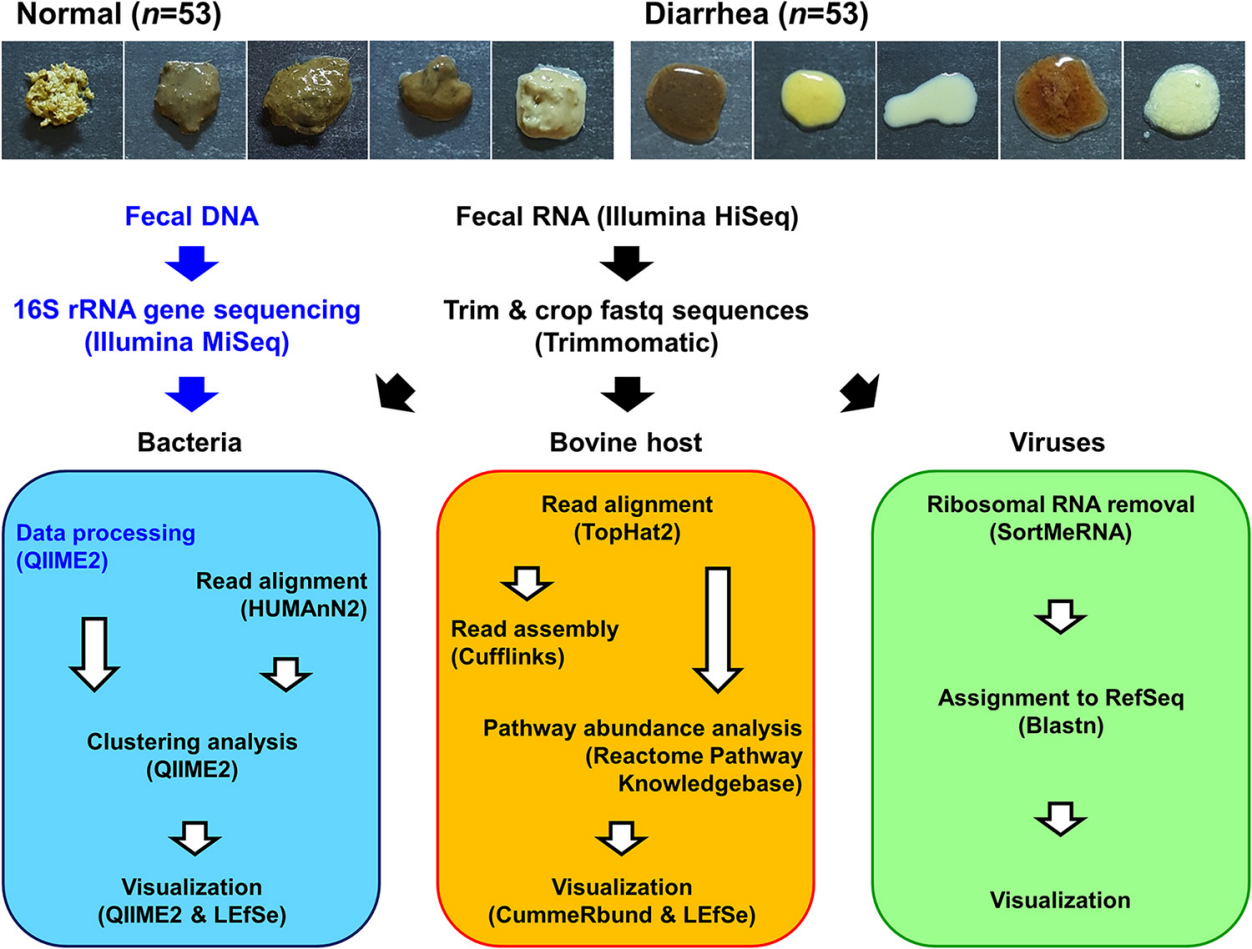
Experimental design for analyzing the calf rectal microbiota and transcriptome. Representative images of the rectal luminal content collected from normal and diarrheic calves (upper panels) and the workflow for sequencing, data processing, and bioinformatics pipeline (lower panels).

An important characteristic of diarrhea, observed in most calves, is the passage of loose stool (20). After the defecating behaviors of young calves were observed, their rectal luminal contents were collected. Stool liquidity, rather than stool frequency, color, or volume, was evaluated, according to the Bristol stool scale, which is frequently used to define diarrhea (21). Samples with Bristol score 7 (watery, no solid pieces, entirely liquid) were classified as the diarrhea group (D, *n *= 53), whereas those with score of ≤6 were classified as the normal group (N, *n *= 53; Fig. 1). No meaningful differences were observed in calf age at sampling between the normal and diarrheic calves. Detailed information on calf age and sex and moisture content of the collected samples, is provided in Table S1.

10.1128/mSystems.00816-20.7 TABLE S1 Calf age and sex and moisture content of the collected samples. Download Table S1, PDF file, 0.2 MB. Copyright © 2021 Whon et al. 2021 Whon et al. https://creativecommons.org/licenses/by/4.0/ This content is distributed under the terms of the Creative Commons Attribution 4.0 International license.

To verify the prevalence of infection with the known allochthonous etiological agents of calf diarrhea in the collected samples, a diagnostic multiplex PCR assay was conducted. In the assay, 14 and 13 primer pairs were used for the detection of viral and bacterial pathogens, respectively (Table 1). According to the assay, several samples were positive for infections with viral (e.g., the group A and C rotaviruses, bovine coronavirus, bovine norovirus, bovine enteric Nebraska-like calicivirus, and bovine viral diarrhea virus) and bacterial (e.g., Clostridium perfringens and shigatoxigenic and enterohemorrhagic Escherichia coli) pathogens (Table 2). Of note, no infecting pathogen was associated exclusively with the diarrheic samples. We next determined the abundance of several toxin genes of pathogenic E. coli (i.e., *stx*_2_ and *eaeA* from shigatoxigenic E. coli and *hlyA* from enterohemorrhagic E. coli) in the calf feces and the surrounding environment (i.e., feed pellet, water, bedding, and maternal milk and feces) by diagnostic multiplex PCR and real-time quantitative PCR. In the samples from the calf environment, the real-time quantitative PCR revealed a meaningful difference in the abundance of toxin genes in the normal and diarrheic samples (i.e., elevated levels of the *stx*_2_ gene in maternal milk and feces of the diarrheic sample and of the *eaeA* gene in the water for the normal sample) (Fig. S1). However, the absolute abundance of pathogenic E. coli seemed to be very low, as evidenced by a lack of signal (detection) in the diagnostic multiplex PCR assay (Table S2). The above-described results suggested that the presence or absence of the known causative pathogens in the samples was not sufficient to explain the etiology of calf diarrhea.

**TABLE 1:** Primer sets for the diagnostic multiplex PCR assay^*a*^

Host	Target gene	Sequence (5′ to 3′)		Reference
		Forward	Reverse	
Viruses				
Group a rotavirus	dsRNA segment 6	GGCTTTTAAACGAAGTCTTC	GGTCACATCCTCTCACTACG	67
Group a rotavirus	VP7	GCCTTTAAAAGCGAGAATTT	GGTCACATCATACAAYTC TA	68
Group B rotavirus	VP7	GGAAATAATCAGAGATG	CTACTCGTTTGGCTCCCTCC	69
Group C rotavirus	VP6	TCAAGAAATGGWATGCAACC	CATAGCMGCTGGTCTWATCA	70
Bovine coronavirus	N	GCAATCCAGTAGTAGAGCGT	CTTAGTGGCATCCTTGCCAA	71
Bovine coronavirus	S	ATGTTTTTGATACTTTTAATTTCC	ACACCAGTAGATGGTGCTAT	70
Bovine torovirus	M	TTCTTACTACACTTTTTGGA	ACTCAAACTTAACACTAG AC	70
Bovine torovirus	N	TAATGGCACTGAAGACTC	ACATAACATCTTACATGG	72
Bovine norovirus	RdRp	AGTTAYTTTTCCTTYTAYGGBGA	AGTGTCTCTGTCAGTCATCTTCAT	73
Bovine enteric Nebraska-like calicivirus	RdRp-MCP	TTTCTAACYTATGGGGAYGAYG	GTCACTCATGTTTCCTTCTCTAAT	73
Bovine nebovirus	Capsid	CCACCATTATCACCAAATTGC	CATAATCAGAATAGAAGGCGC	74
Bovine viral diarrhea virus	Bsteii	GATTTCAAGGGGACTTTTTT	ACATCTCCTACTAAGTAGTA	75
Bovine viral diarrhea virus	Bvdv1 genotype	GTAGTCGTCAGTGGTTCG	GCCATGTACAGCAGAGAT	75
Bovine viral diarrhea virus	Polyprotein	ACAAACATGGTTGGTGCAACTGGT	CAGACATATTTGCCTAGGTTCCA	76
Bacteria				
Clostridium perfringens	16S rRNA gene	AAAGATGGCATCATCATTCAAC	TACCGTCATTATCTTCCCCAAA	77
Clostridium perfringens	Alpha-toxin genes	GCTAATGTTACTGCCGTTGACC	TCTGATACATCGTGTAAG	77
Salmonella enterica	Sefb	AGATTGGGCACTACACGTGT	TGTACTCCACCAGGTAATTG	78
Salmonella enterica Typhimurium	Rfbj	CCAGCACCAGTTCCAACTTGATAC	GGCTTCCGGCTTTATTGGTAAGCA	79
Enterotoxigenic Escherichia coli	K99	GCTATTAGTGGTCATGGCACTGTAG	TTTGTTTTGGCTAGGCAGTCATTA	80
Enterotoxigenic Escherichia coli	LT1	GCTGACTCTAGACCCCCAG	TGTAACCATCCTCTGCCGGA	81
Enterotoxigenic Escherichia coli	LT2	ATATCATTTTCTGTTTCAGCAAA	CAATAAAATCATCTTCGCTCATG	82
Enterotoxigenic Escherichia coli	ST1	TCCCCTCTTTTAGTCAGTCAACTG	GCACAGGCAGGATTACAACAAAGT	83
Enterotoxigenic Escherichia coli	ST2	CTGTGTGAACATTATAGACAAATA	ACCATTATTTGGGCGCCAAAG	81
Shigatoxigenic Escherichia coli	stx1	GACTGCAAAGACGTATGTAGATTCG	ATCTATCCCTCTGACATCAACTGC	84
Shigatoxigenic Escherichia coli	stx2	ATTAACCACACCCCACCG	GTCATGGAAACCGTTGTCAC	84
Shigatoxigenic Escherichia coli	eaeA	GACCCGGCACAAGCATAAGC	CCACCTGCAGCAACAAGAGG	85
Enterohemorrhagic Escherichia coli	hlyA	GCATCATCAAGCGTACGTTCC	AATGAGCCAAGCTGGTTAAGCT	85

a Abbreviations: dsRNA segment 6, double-stranded RNA genome segment 6; VP, viral protein; N, nucleocapsid protein; S, S glycoprotein; M, membrane protein; RdRp, RNA-dependent RNA polymerase; MCP, major capsid protein; BstEII, restriction enzyme BstEII; sefB, chaperone protein SefB coding gene; rfbJ, CDP-abequose synthase coding gene; K99, K99 region 1 gene; LT1, heat-labile enterotoxin type 1 A subunit; LT2, heat-labile enterotoxin type 2; ST1, heat-stable enterotoxin 1; ST2, heat-stable enterotoxin 2; stx1, Shiga toxin type 1; stx2, Shiga toxin type 2; eaeA, enterohemorrhagic E. coli O157:H7-specific intimin; hlyA, plasmid-encoded enterohemolysin.

**TABLE 2:** Results of the diagnostic multiplex PCR^*a*^*^,^*^*b*^

Host	Target gene	No. of PCR positive samples for:		*P* value
		Normal (*n *= 53)	Diarrhea (*n *= 53)	
Viruses				
Group a rotavirus	dsRNA segment 6	3	1	0.1574
Group a rotavirus	VP7	0	0	
Group B rotavirus	VP7	0	0	
Group C rotavirus	VP6	1	4	0.0870
Bovine coronavirus	N	1	1	0.4947
Bovine coronavirus	S	0	0	
Bovine torovirus	M	0	0	
Bovine torovirus	N	0	0	
Bovine norovirus	RdRp	2	1	0.2837
Bovine enteric Nebraska-like calicivirus	RdRpMCP	3	1	0.1574
Bovine nebovirus	Capsid	0	0	
Bovine viral diarrhea virus	BstEII	0	0	
Bovine viral diarrhea virus	BVDV1 genotype	3	1	0.1574
Bovine viral diarrhea virus	Polyprotein	0	0	
Bacteria				
Clostridium perfringens	16S rRNA gene	16	24	0.0557
Clostridium perfringens	Alpha-toxin genes	5	3	0.2342
Salmonella enterica	sefb	0	0	
Salmonella enterica Typhimurium	rfbJ	0	0	
Enterotoxigenic Escherichia coli	K99	0	0	
Shigatoxigenic Escherichia coli	stx1	15	10	0.1283
Shigatoxigenic Escherichia coli	stx2	13	18	0.1449
Shigatoxigenic Escherichia coli	eaeA	10	8	0.3050
Enterohemorrhagic Escherichia coli	hlyA	18	18	0.4985

a Abbreviations: dsRNA segment 6, double-stranded RNA genome segment 6; VP, viral protein; N, nucleocapsid protein; S, S glycoprotein; M, membrane protein; RdRp, RNA-dependent RNA polymerase; MCP, major capsid protein; BstEII, restriction enzyme BstEII; sefB, chaperone protein SefB coding gene; rfbJ, CDP-abequose synthase coding gene; K99, K99 region 1 gene; stx1, Shiga toxin type 1; stx2, Shiga toxin type 2; eaeA, enterohemorrhagic E. coli O157:H7-specific intimin; hlyA, plasmid-encoded enterohemolysin.

b The data were analyzed using the nonparametric Mann-Whitney *U* test (one-tailed).

10.1128/mSystems.00816-20.1 FIG S1 Quantitative analysis of toxin genes of pathogenic E. coli in the normal and diarrheic samples. The abundance of toxin genes (Shiga toxin type 2 [*stx2*], enterohemorrhagic E. coli O157:H7-specific intimin [*eaeA*], and plasmid-encoded enterohemolysin [*hlyA*]) in the normal and diarrheic samples was determined using real-time quantitative PCR. The feces, feed pellet, water, bedding, and maternal milk and feces were collected from normal (N, *n *= 6) and diarrheic calves (D, *n *= 5). The values were normalized to the abundance of the bacterial 16S rRNA gene and are presented as relative amounts. The data were analyzed using the nonparametric Mann-Whitney *U* test (one-tailed; *, *P* < 0.05; **, *P* < 0.01; ***, *P < *0.001). Data are shown as mean ± SEM. Download FIG S1, JPG file, 0.4 MB. Copyright © 2021 Whon et al. 2021 Whon et al. https://creativecommons.org/licenses/by/4.0/ This content is distributed under the terms of the Creative Commons Attribution 4.0 International license.

10.1128/mSystems.00816-20.8 TABLE S2 Results of the diagnostic multiplex PCR analysis of samples from the calf environment. Download Table S2, PDF file, 0.2 MB. Copyright © 2021 Whon et al. 2021 Whon et al. https://creativecommons.org/licenses/by/4.0/ This content is distributed under the terms of the Creative Commons Attribution 4.0 International license.

### Rectal bacterial metataxonomic analysis reveals increased abundance of the family *Enterobacteriaceae* in diarrheic calves.

**FIG 2:**
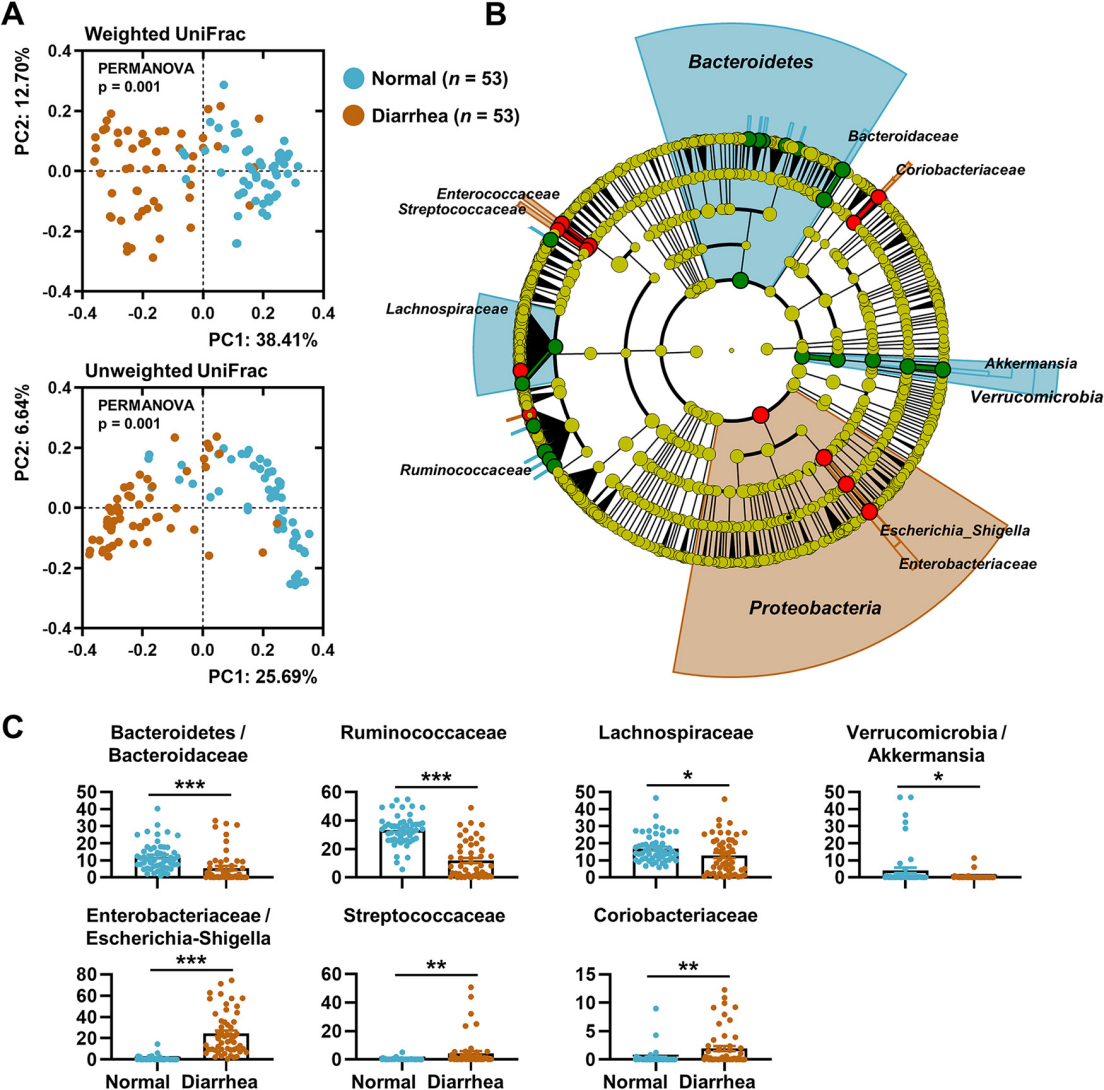
Rectal bacterial profiles of normal and diarrheic calves. (A) PCoA of the rectal bacterial 16S rRNA sequences based on the weighted and unweighted UniFrac distance matrices. Data from the normal and diarrheic groups (*n *= 53 for each) are shown. (B) The abundance patterns of bacterial taxa in each group analyzed using the LEfSe circular cladogram. The discriminant taxa for each group are denoted in different colors. (C) Relative abundance of the discriminant taxa in ASV feature tables for each group presented as bar graphs. The data were analyzed using the multiple *t* test. Correction for multiple comparisons was made using the false-discovery rate (FDR; threshold of 0.05). *, adjusted *P* < 0.05; **, *P* < 0.01; ***, *P* < 0.001. Data are shown as mean ± SEM.

To test whether the diarrheic gut harbored a dysbiotic bacterial microbiota, 16S rRNA gene profiles from the rectal luminal samples were investigated (*n *= 53 each for the normal and diarrhea groups) (Fig. 1). On average, 177,023 ± 56,705 paired-end reads were obtained for each sample. Principal-coordinate analysis (PCoA) of both the weighted and unweighted UniFrac distance matrices revealed separate clusters of data points by group (permutational multivariate analysis of variance [PERMANOVA], *P = *0.001; Fig. 2A). The linear discriminant analysis effect size (LEfSe) (22) circular cladogram indicated that the phyla *Bacteriodetes* and *Proteobacteria* were the discriminant taxa of the normal and diarrheic rectal samples, respectively (Fig. 2B, Fig. S2). The relative abundances of the taxa given by the LEfSe in the amplicon sequence variant (ASV) feature tables were compared. Sequences assigned to the families *Bacteroidaceae*, *Ruminococcaceae*, and *Lachnospiraceae*, and the genus *Akkermansia* were significantly enriched in normal rectal samples, whereas the diarrheic samples were characterized by a high abundance of sequences assigned to *Escherichia-Shigella* and the families *Streptococcaceae* and *Coriobacteriaceae*, with meaningful significance (multiple *t* test, adjusted *P < *0.05; Fig. 2C).

10.1128/mSystems.00816-20.2 FIG S2 Composition and structure of the rectal bacterial community in normal, diarrheic, and intermittently diarrheic calves. (A) Relative abundance of the rectal bacterial 16S rRNA sequences in normal (*n *= 53) and diarrheic (*n *= 53) calves at the phylum level. (B) Relative abundance of the family *Enterobacteriaceae* members among the phylum *Proteobacteria* members. (C) Relative abundance of the rectal bacterial 16S rRNA sequences in calves with repeated normal diarrhea (ND, 23 samples from 5 calves) at the phylum level. (D) Relative abundance of the family *Enterobacteriaceae* members among the phylum *Proteobacteria* members. Download FIG S2, JPG file, 0.7 MB. Copyright © 2021 Whon et al. 2021 Whon et al. https://creativecommons.org/licenses/by/4.0/ This content is distributed under the terms of the Creative Commons Attribution 4.0 International license.

### Temporal variation in the abundance of *Enterobacteriaceae* is positively correlated with the incidence of diarrhea.

**FIG 3:**
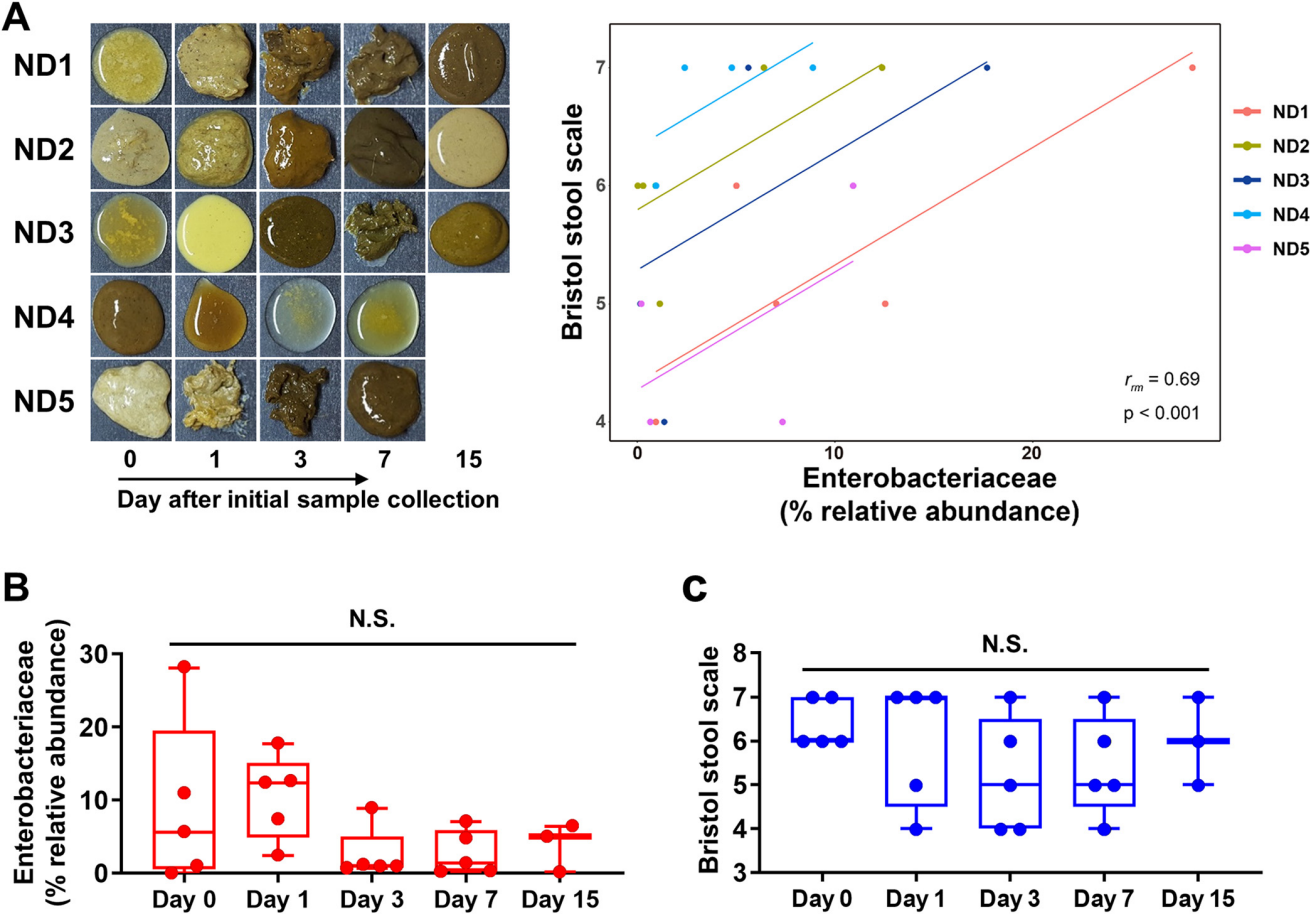
The correlation coefficient analysis of the gut *Enterobacteriaceae* and the incidence of diarrhea in intermittently diarrheic calves. Rectal luminal contents were collected from calves with repeated normal diarrhea (ND, 23 samples from 5 calves). (A) Images of the rectal luminal content of the ND samples (left). The repeated measures correlation was calculated based on the relative abundance of the family *Enterobacteriaceae* (*x* axis) and the Bristol score (*y* axis) of the collected samples (right). (B and C) The relative abundance of the family *Enterobacteriaceae* (B) and the Bristol score of the samples (C) were categorized by the sample collection time. The data were analyzed by ANOVA followed by Tukey’s *post hoc* test (N.S., not significant). Data are shown as mean ± SEM.

Several autochthonous bacterial species (but not the exogenous enteropathogens) belonging to the phylum *Proteobacteria* are regarded as the natural microbiota of the mammalian gut, because they are commonly found in the gut of terrestrial animals (7, 23, 24). Accordingly, sequences assigned to *Proteobacteria* (mostly from the family *Enterobacteriaceae*) were identified in all rectal samples regardless of sample type (Fig. S2A and B). The possible role of the temporal changes in abundance (rather than the presence or absence) of *Enterobacteriaceae* in the progression of calf diarrhea was then tested. After close evaluation of calf defecating behaviors, rectal luminal samples were collected from five intermittently diarrheic calves (i.e., animals with repeated normal diarrhea, ND). The bacterial microbiota from the collected samples was longitudinally profiled by metataxonomic analysis. A mean of 174,913 ± 28,121 paired-end reads was obtained for each sample. A dynamic fluctuation of several bacterial taxa was observed, including the phyla *Firmicutes*, *Bacteroidetes*, and *Proteobacteria*, within the individual calves (Fig. S2C and D). Of note, a significantly positive correlation was observed between the intrasample variations of the relative abundance of *Enterobacteriaceae* and the Bristol score (repeated measures correlation [25] *r_rm_* = 0.69, *P < *0.001; Fig. 3A). To rule out the possibility that the diarrheic symptoms were alleviated spontaneously with calf aging, we categorized the samples by sample collection time and conducted a time-series statistical analysis. We observed no meaningful differences in the relative abundance of *Enterobacteriaceae* (Fig. 3B) or the Bristol score associated with the sample collection time (Fig. 3C). Collectively, the above-described bacterial metataxonomic analysis of the diarrheic gut suggested that gut dysbiosis exemplified by an abnormal increase in the abundance of *Enterobacteriaceae* is highly likely to trigger the diarrheic symptoms in young calves.

### Metatranscriptomics reveals robust aerobic respiration of rectal bacterial microbiota in diarrheic calves.

To better understand the changes in the transcriptional landscape of the intestine during the progression of diarrhea, we selected 18 samples in numerical order from the 106 normal and diarrheic rectal luminal samples and conducted RNA-Seq-based transcriptomics analysis. Illumina HiSeq paired-end sequencing generated a similar number of raw rectal cDNA sequences across the samples (mean, 48,198,713 ± 4,700,228 reads). The tripartite transcriptional interaction among the gut bacteria, bovine host, and viruses was subsequently evaluated based on these raw reads (Fig. 1).

**FIG 4:**
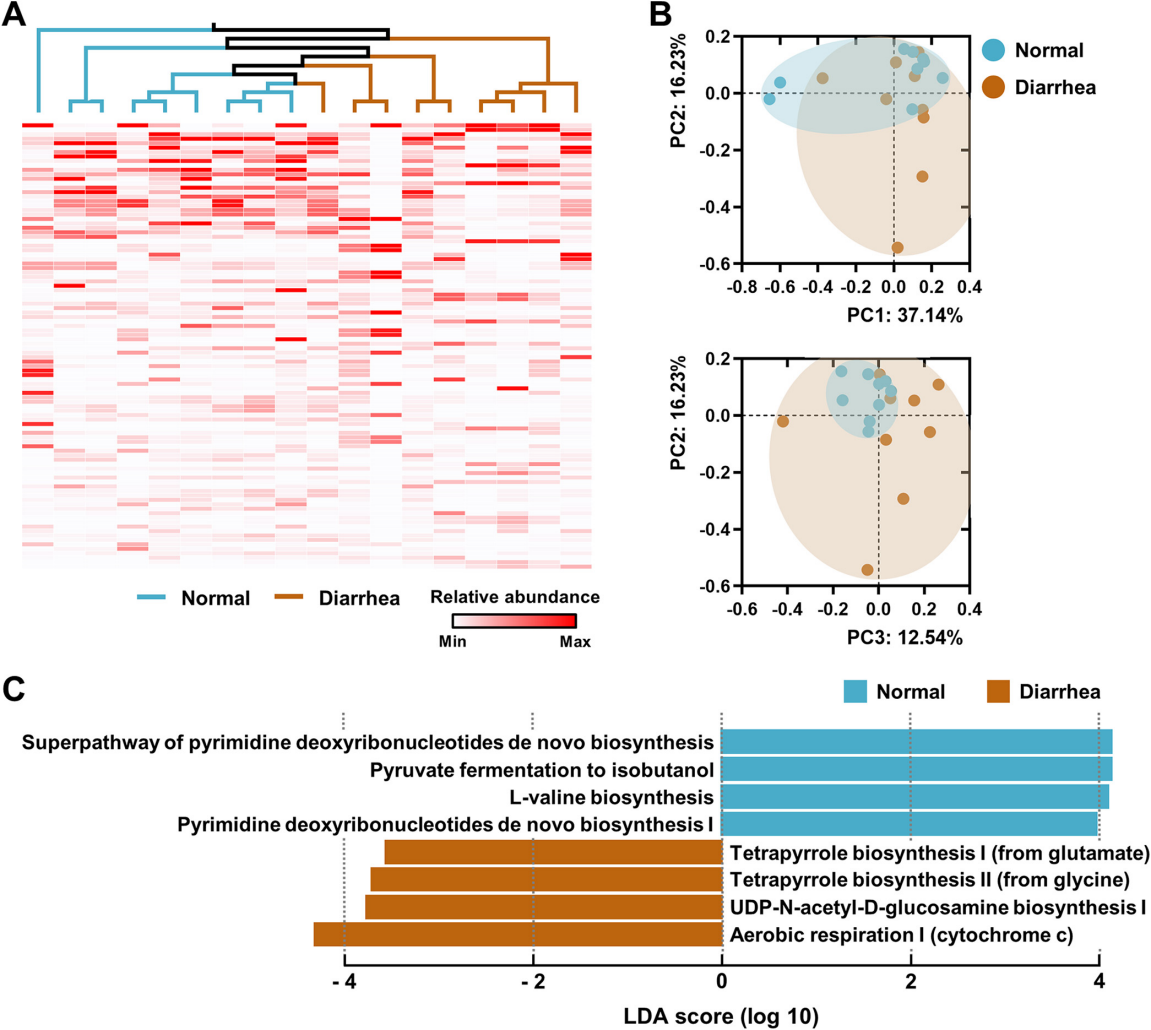
Metatranscriptomic profiles of the rectal microbiome of normal and diarrheic calves. Normal (*n *= 9) and diarrheic (*n *= 9) rectal metatranscripts generated by RNA-Seq were functionally profiled using the HUMAnN2 pipeline. (A) The genes abundantly expressed in samples (the top 100 among the 121,568 assigned genes) were clustered using the UPGMA dendrogram based on the abundance-weighted Jaccard distance (abund_jaccard). The relative abundance of the expressed genes is presented as a heatmap. (B and C) At the pathway level, pathway abundances in the samples were clustered using the abund_jaccard-based PCoA (B), and the discriminant pathways for each group were determined using the LEfSe (C).

First, global expression patterns of rectal bacterial genes in normal and diarrheic calves were assessed using the HUMAnN2 metatranscriptomics approach. The unweighted pair group method using average linkages (UPGMA) dendrogram (based on the abundance-weighted Jaccard distance [abund_jaccard]) combined with a heatmap analysis of abundantly expressed genes (the top 100 among 121,568 assigned genes) revealed relatively commonly shared profiles of the highly expressed genes in normal calves (Fig. 4A). At the pathway level, a PC2 versus PC3 plot of PCoA based on the abund_jaccard matrix confirmed rigid clustering of pathway abundance plots in the normal group (Fig. 4B). In diarrheic calves, however, the gene family and pathway abundance profiles represented the features of shared transcriptional patterns that were less robust than those in the normal group, and the plots were distantly scattered from those of the normal group (Fig. 4A and B).

A specific pattern of pathway abundance in bacterial metatranscriptomes within each group was identified using the LEfSe method. The effect size estimations of LEfSe indicated that pyrimidine metabolisms, pyruvate fermentation to isobutanol, and l-valine biosynthesis were the discriminant pathways for the bacterial microbiota in normal calves, whereas aerobic respiration and biosynthesis of UDP-*N-*acetyl-d-glucosamine (UDP-GlcNAc) and tetrapyrrole were the discriminant pathways for the diarrheic calves (Fig. 4C, Fig. S3A). In bacteria, UDP-GlcNAc is a precursor of the cell wall peptidoglycan, the lipopolysaccharide, and the enterobacterial common antigen (26). Growing recent evidence indicates that the metabolism and conversion of GlcNAc to UDP-GlcNAc play important roles in bacterial pathogenesis (27, 28). Next, gene family abundance was stratified at the community level to determine the contributions from known bacterial species. Transcripts from the genus *Escherichia* were predominant in several diarrheic guts (Fig. S3B). These diarrhea-associated metatranscription profiles suggested elevated levels of the intestinal oxygen, perhaps available to the aerobic and/or facultative microbes (e.g., the genus *Escherichia*) as a terminal electron acceptor. They also indicated that the virulence potential of the dysbiotic bacterial microbiome, followed by abundant aerobic respiration and/or oxygen exposure, may be increased. Taken together, the above-described metataxonomic and metatranscriptomic analyses of the diarrheic microbiome suggested that the *Enterobacteriaceae* taxa are active at the DNA (cell abundance) and RNA (gene expression) levels.

10.1128/mSystems.00816-20.3 FIG S3 Discriminant pathways in the rectal metatranscriptomes of normal and diarrheic calves (A) and transcriptomic contribution of known bacterial species in the normal and diarrheic calf rectal transcriptomes (B). (A) The normal (*n *= 9) and diarrheic (*n *= 9) rectal metatranscripts generated by RNA-Seq were functionally profiled using the HUMAnN2 pipeline. Relative abundances of the discriminant pathways for each group are presented as bar graphs. The data were analyzed using the nonparametric Mann-Whitney *U* test (one-tailed; *, *P* < 0.05; **, *P* < 0.01; ***, *P* < 0.001). Data are shown as mean ± SEM. (B) The normal (*n *= 9) and diarrheic (*n *= 9) rectal metatranscripts generated by RNA-Seq were functionally profiled using the HUMAnN2 pipeline. The community functional profiles stratified according to the relative abundance of the known and unclassified organisms are presented. Download FIG S3, JPG file, 0.5 MB. Copyright © 2021 Whon et al. 2021 Whon et al. https://creativecommons.org/licenses/by/4.0/ This content is distributed under the terms of the Creative Commons Attribution 4.0 International license.

### The bovine host transcriptome links elevated sulfur metabolism, innate immunity, and gut motility with diarrhea.

**FIG 5:**
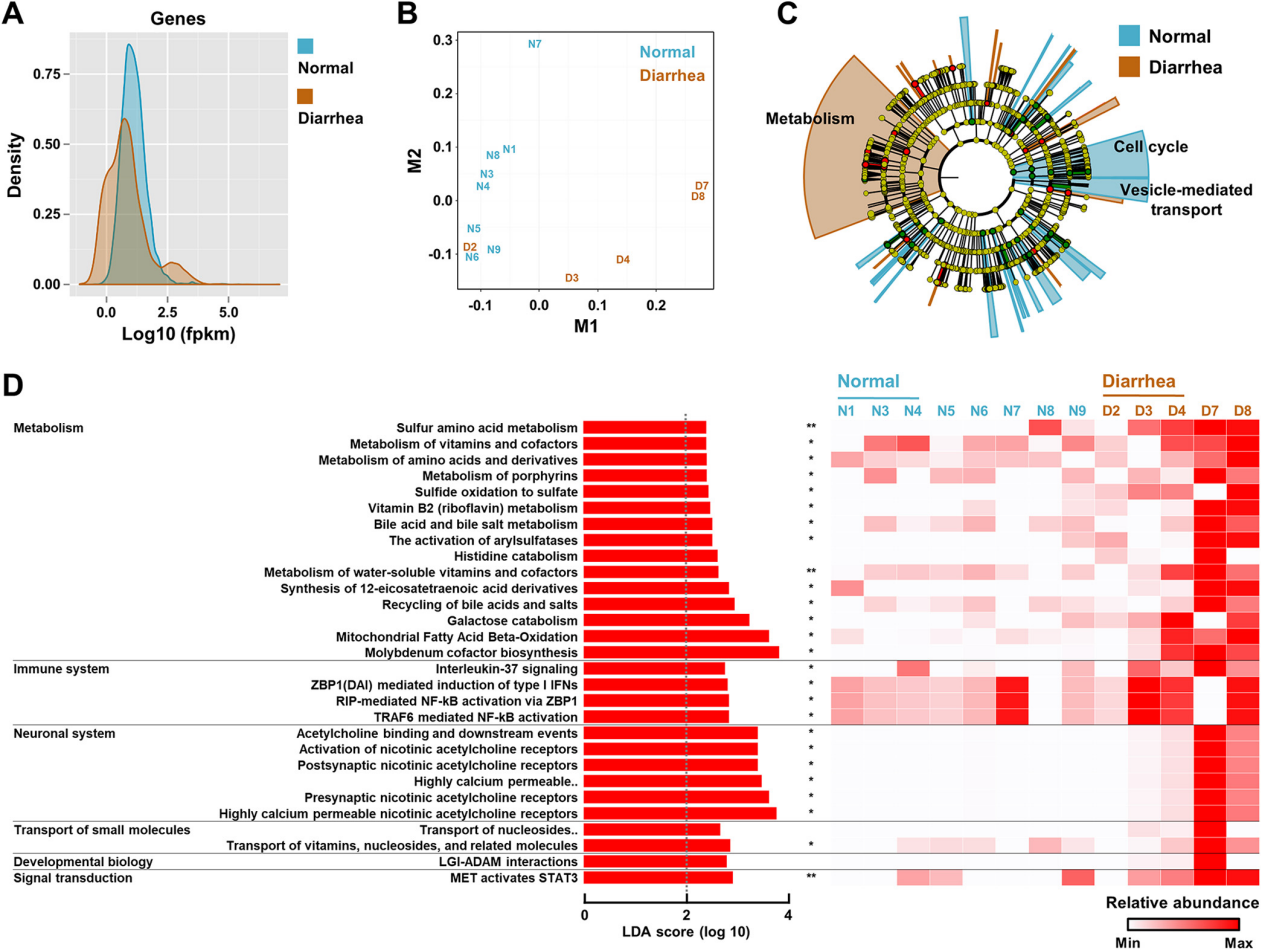
Host rectal transcriptomic profiles in the normal and diarrheic calves. Rectal transcripts generated by RNA-Seq were analyzed according to the Tuxedo protocol (the TopHat2, Cufflink, and CummeRbund packages). Host transcriptomes from samples with an over 0.05% mapping rate of the processed reads to the bovine genome were compared (*n *= 8 and 5 for the normal and diarrheic groups, respectively). (A and B) Global difference in the fragments per kilobase million (FPKM) scores (A) and dimensionality reduction between groups (B), presented as a density plot with log_10_ values and a multidimensional scaling plot, respectively. (C) The bovine genome-mapped reads were mapped to the Reactome Pathway Knowledgebase, and the results were visualized using the LEfSe circular cladogram. The discriminant pathways for each group are denoted by different colors. (D) Specific patterns of the discriminant pathways were identified by the LEfSe method. The different pathway abundances are presented using the LDA score and a heatmap. Abbreviations: highly calcium permeable, highly calcium permeable postsynaptic nicotinic acetylcholine receptors; transport of nucleosides, transport of nucleosides and free purine and pyrimidine bases across the plasma membrane. The data were analyzed using the multiple *t* test. Corrections for multiple comparisons were made using the false-discovery rate (FDR; threshold of 0.05). *, adjusted *P* < 0.05; **, *P* < 0.01. Data are shown as mean ± SEM.

The rectal luminal contents are a proxy for assessing the host gut transcripts, because the intestinal epithelial cells are constantly shed into the gut lumen as part of epithelial homeostasis (29, 30). Host transcriptomes in samples with an over 0.05% (>20,000 reads) mapping rate of the processed sequences to the bovine genome were compared in normal (*n *= 8) and diarrheic calves (*n *= 5). A density plot generated by the CummeRbund package revealed a global difference in the fragments per kilobase million (FPKM) scores of the normal and diarrheic rectal transcriptomes (Fig. 5A). Similarly, multidimensional scaling analysis resulted in separate clusters of data points according to groups (except for sample D2; Fig. 5B), supporting the change of the transcriptional profile of the bovine host in response to diarrhea.

Detailed functional relationships were then inferred from the gene expression profiles. To this end, the bovine genome-mapped reads were annotated using the Reactome Pathway Knowledgebase, and the results were visualized using the LEfSe method. The pathway abundance profiles of the host rectal transcriptomes represented cell cycle-weighted and metabolism-weighted pathway abundances for the normal and diarrheic groups, respectively (Fig. 5C). In the diarrheic calf transcriptome, the following cellular pathways were significantly enriched (multiple *t* test, adjusted *P < *0.05); pathways related to (i) sulfur metabolism (e.g., sulfur amino acid metabolism, sulfide oxidation to sulfate, the activation of arylsulfatases, and molybdenum cofactor biosynthesis), (ii) innate immunity (e.g., ZBP1-mediated induction of type I interferons [IFNs], and RIP- and TRAF6-mediated NF-κB activations), and (iii) the neuronal system (e.g., activation of nicotinic acetylcholine receptors) (Fig. 5D). The presynaptic nicotinic acetylcholine receptors of the enteric nervous system play an important role in the regulation of gut motility (31). Taken together, the described intertranscriptomic relationship between the gut bacteria and the bovine host suggested that the diarrheic gut constitutes a distinct environmental niche (as exemplified by elevated sulfur metabolism, immune responses, and gut motility), wherein the conditions favor the growth of aerobic and/or facultative microbes, such as the genus *Escherichia*.

### Viral transcriptomics analysis reveals a high abundance of the *Enterobacteriaceae*-infecting bacteriophages in the diarrheic gut.

The rectal luminal transcripts represent the genomic components of RNA viruses and transcripts of DNA viruses. A stringent assignment of the rectal transcripts using the viral database enabled profiling of the gut virome (i.e., viral community) in the normal and diarrheic calves (Fig. 1). The assigned viruses were classified into three categories according to their infecting host (i.e., mammal-, plant-, and bacterium-infecting viruses) (Table 3). In the mammalian virus category, the sequences assigned to rotavirus, calicivirus, and Newbury agent-1 virus were most abundant across the samples. The distributions of sequences assigned to the known etiological viruses were not exclusively weighted to the diarrheic samples. These findings, combined with the results of the multiplex PCR assay (Table 2), were in agreement with previous reports that described a weak relationship between the diarrhea-associated microbiological agents and the onset of diarrhea (5, 32).

**TABLE 3:** Viral transcriptomic profiles of the normal and diarrheic rectal microbiomes^*a*^

Host	Assigned virus	Read counts for normal group									Read counts for diarrhea group									*P* value
		N1	N2	N3	N4	N5	N6	N7	N8	N9	D1	D2	D3	D4	D5	D6	D7	D8	D9	
Mammals	Rotavirus	1	0	0	0	39	1	1	0	3	0	0	2	1	311	0	0	1,479	0	
Mammals	Calicivirus	67	0	4	0	0	0	0	1	1,314	1	0	0	0	0	0	0	0	3	
Mammals	Newbury agent 1 virus	59	1	2	0	0	1	0	1	741	0	0	0	0	1	2	1	0	0	
Mammals	Bovine astrovirus	0	0	0	0	1	30	0	2	4	0	0	0	62	0	0	0	0	0	
Mammals	Norovirus	0	0	0	0	0	0	0	0	88	0	0	0	0	0	0	1	0	0	
Mammals	Bovine hungarovirus	0	5	0	3	0	0	0	0	0	0	4	0	0	0	0	2	0	20	
Mammals	Bovine kobuvirus	0	1	0	0	0	2	0	10	0	0	0	0	0	1	0	0	12	1	
Mammals	Breda virus	0	0	0	0	0	0	0	0	0	1	0	0	0	0	0	23	0	0	
Mammals	BeAn 58058 virus	0	0	1	1	0	3	1	1	0	0	0	2	2	1	1	3	0	2	
Mammals	Porcine torovirus	0	0	0	0	0	0	0	0	0	0	0	0	0	0	0	18	0	0	
Mammals	Goat torovirus	0	0	0	0	0	0	0	0	0	0	0	0	0	0	0	13	0	0	
Mammals	Bovine herpesvirus	0	0	1	0	1	6	0	0	0	0	1	3	0	0	0	0	0	0	
Mammals	Human endogenous retrovirus	2	1	1	0	1	1	0	0	1	0	0	0	1	0	0	1	2	0	
Mammals	Hepatitis B virus	0	0	0	0	0	3	0	0	0	0	1	0	1	0	0	0	0	1	
Mammals	Enterovirus	0	0	0	0	0	1	0	0	0	0	1	1	0	0	0	0	0	0	
Mammals	Bat picornavirus	0	0	0	0	0	0	2	0	0	0	0	0	0	0	0	0	0	0	
Mammals	Porcine astrovirus	1	0	0	0	0	0	0	0	0	0	0	0	0	0	0	0	0	0	
Mammals	Pestivirus	0	0	0	0	0	0	0	0	1	0	0	0	0	0	0	0	0	0	
Mammals	Y73 sarcoma virus	0	0	0	1	0	0	0	0	0	0	0	0	0	0	0	0	0	0	
Mammals	Yak enterovirus	0	0	0	0	0	0	0	0	0	0	1	0	0	0	0	0	0	0	
Plants	Sweet potato feathery mottle virus	0	0	0	0	0	0	0	0	0	2	0	0	0	0	0	0	0	0	
Plants	Pepper mild mottle virus	0	0	0	0	0	1	0	0	0	0	0	0	0	0	0	0	0	0	
Bacteria	Enterobacteria phage	0	0	7	0	0	3	0	3	9	3	253	113	3	73	39	38	15	37	0.001
Bacteria	Stx2 converting phage	0	0	0	0	4	0	1	0	0	0	52	1	0	5	12	17	0	5	0.016
Bacteria	*Escherichia* phage	0	0	2	1	1	0	0	0	0	0	8	51	0	7	10	0	1	1	0.044
Bacteria	*Salmonella* phage	0	0	1	0	1	0	1	0	0	0	11	0	0	6	7	4	2	1	0.026
Bacteria	Bacteriophage RB32	0	0	0	0	0	0	0	0	0	0	6	0	1	14	1	1	0	5	
Bacteria	*Lactobacillus* prophage	0	0	2	3	0	0	2	0	2	0	0	4	1	0	5	0	0	0	
Bacteria	Phage cdtI DNA	0	0	0	0	0	0	0	0	0	0	7	0	0	0	4	0	0	1	
Bacteria	*Bacteroides* phage	0	0	0	0	0	0	0	0	11	0	0	0	0	0	0	0	0	0	
Bacteria	*Streptococcus* phage	0	1	0	0	3	0	0	0	1	0	0	0	0	5	0	1	0	0	
Bacteria	*Yersinia* phage	0	0	2	0	0	0	0	0	0	0	0	2	0	3	1	0	0	1	
Bacteria	*Lactobacillus* phage	0	0	0	0	0	0	0	0	1	0	0	0	0	0	5	0	0	0	
Bacteria	*Erwinia* phage	0	0	0	0	0	0	0	0	0	0	0	0	0	1	0	3	0	0	
Bacteria	Lactobacillus johnsonii prophage	0	0	2	0	0	0	0	0	2	0	0	0	0	0	0	0	0	0	
Bacteria	Bacteriophage WPhi	0	0	0	0	0	0	0	0	0	0	0	0	0	1	0	2	0	0	
Bacteria	Stx1 converting phage	0	0	0	0	0	0	0	0	0	0	3	0	0	0	0	0	0	0	
Bacteria	*Clostridium* phage	0	0	0	0	0	0	0	0	0	0	0	1	1	0	0	0	0	0	
Bacteria	Bacteriophage 186	0	0	0	0	0	0	0	0	0	0	0	0	0	0	0	1	0	0	
Bacteria	Bacteriophage HK022	0	0	0	0	0	0	0	0	0	0	1	0	0	0	0	0	0	0	
Bacteria	*Chlamydia* phage	0	0	0	0	0	0	0	0	0	0	0	0	1	0	0	0	0	0	
Bacteria	*Haemophilus* phage	0	0	0	0	0	1	0	0	0	0	0	0	0	0	0	0	0	0	
Assigned reads (95%id + 90% qcov)		130	9	25	9	51	53	8	18	2,178	7	349	180	74	429	87	129	1,511	78	0.047
Total reads (R1+R2)		95,640	84,840	308,984	565,492	435,548	3,142,388	172,298	995,482	1,274,920	388,702	382,930	1,343,134	2,207,744	81,836	219,518	382,370	540,896	111,362	

a The data were analyzed using the nonparametric Mann-Whitney *U* test (one-tailed).

Interestingly, however, a meaningful difference was observed in the abundance of several members of the bacteriophage population (e.g., *Enterobacteria* phage, *stx*_2_ converting phage, *Escherichia* phage, and *Salmonella* phage) in the normal and diarrheic samples (Mann-Whitney *U* test, *P < *0.05; Table 2). The bacteriophages listed above are DNA viruses that infect host *Enterobacteriaceae* species, and their abundance was highly weighted to the diarrheic samples. Considering that RNA-Seq was used to capture the rectal luminal RNA, the viral transcription data suggested that the abundant *Enterobacteriaceae*-infecting bacteriophages in the diarrheic gut were “transcriptionally active.”

### The diarrheic gut favors the induction of the lytic cycle of the temperate gut bacteriophages.

**FIG 6:**
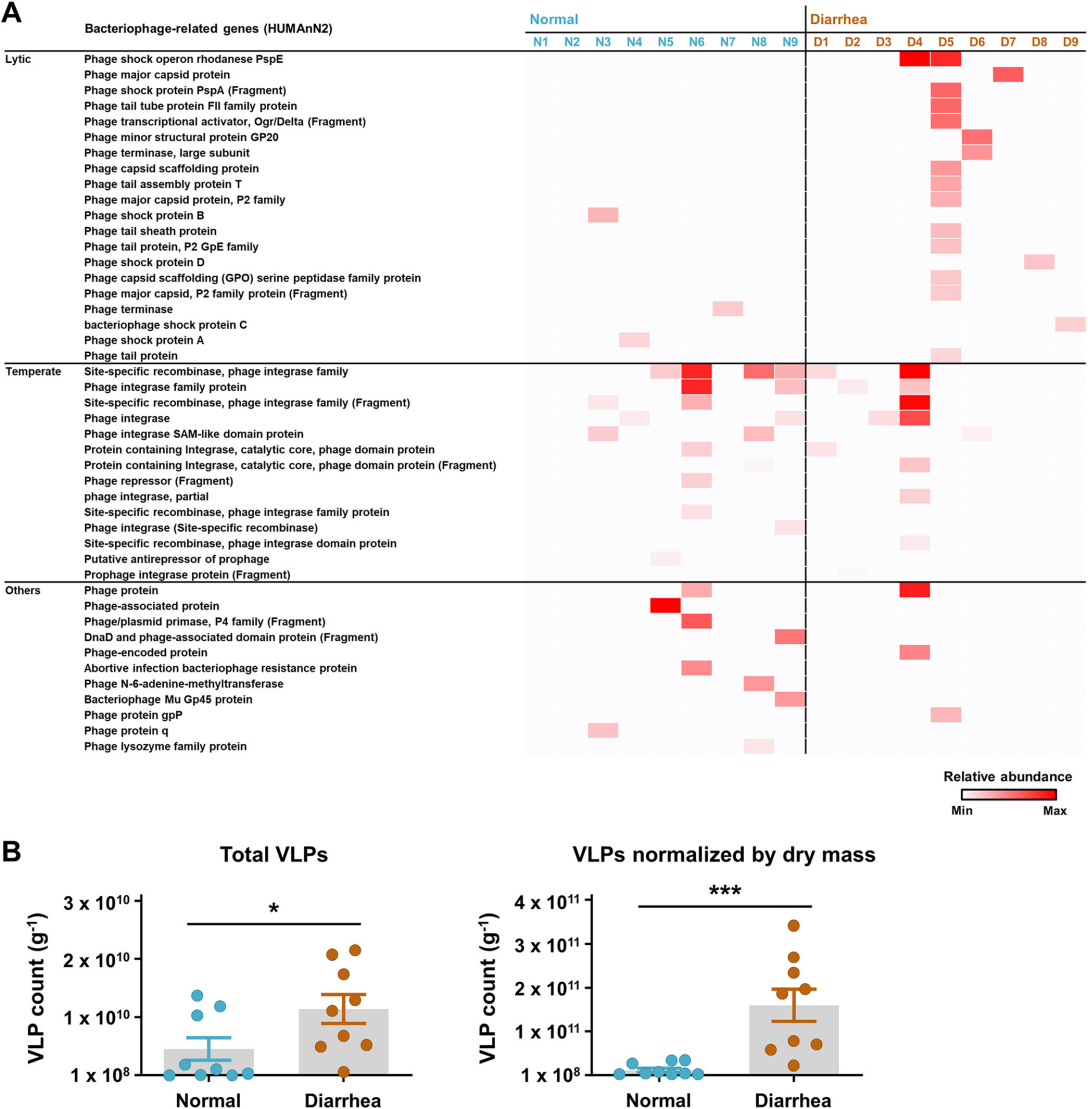
Lytic and temperate features of gut bacteriophages in the normal and diarrheic calves. (A) The bacteriophage-related genes were retrieved from the rectal bacterial metatranscription data (HUMAnN2) of normal and diarrheic calves (*n *= 9 for each) and categorized according to their encoding potential (e.g., lytic and temperate). The relative abundance of the retrieved genes is presented as a heatmap. (B) Numbers of rectal luminal virus-like particles (VLPs) stained with SYBR gold for DNA viruses in the normal and diarrheic samples. Total VLPs and VLPs normalized to the rectal luminal dry mass are shown. The data were analyzed using the nonparametric Mann-Whitney *U* test (one-tailed; *, *P* < 0.05; **, *P* < 0.01; ***, *P* < 0.001). Data are shown as mean ± SEM.

Bacteriophages are abundant in the mammalian gut, and most of them are characterized by a lysogenic life cycle (18, 33). Recent accumulating evidence suggests that the switch of the gut bacteriophage replication cycle from a lysogenic to lytic cycle leads to horizontal gene transfer in the host bacterial population, enabling diversification of the population gene pool, including additional virulence genes and/or antibiotic resistance genes, and rendering the animal host gut more diarrheagenic (13). Accordingly, the temperate and lytic features of gut bacteriophages were deduced from the bacterial metatranscript data since both prophage induction and infection with an exogenous free phage affect the host bacterial RNA metabolism. Bacteriophage-related genes were retrieved from the gene family abundance data (HUMAnN2) and categorized based on the encoded potential (e.g., genes related to structural and shock proteins, and the terminase, were in the “lytic” category, whereas genes related to the recombinase and integrase were in the “temperate” category). In the diarrheic group, five out of nine samples possessed more lytic than temperate features (Fig. 6A). In the normal group, the majority of samples had more temperate than lytic features. However, the normal group also possessed several replication-related (e.g., for the phage/plasmid primase and DnaD) and lysis-related transcripts (e.g., for the abortive infection bacteriophage resistance protein and phage lysozyme family protein) in the “others” category. These transcripts were indicative of an active lytic cycle, suggesting that the gut bacterial metatranscript data only partly supported the notion of abundant temperate phageome in the normal calves.

We hypothesized that the number of enteric bacteriophages would be increased in response to a shift from the lysogenic to lytic replication cycle in the diarrheic calf. To verify this, we isolated virus-like particles (VLPs) from the normal and diarrheic samples (*n *= 9 for each), stained them with SYBR gold for DNA viruses (mostly bacteriophages), and counted the VLPs under an epi-fluorescence microscope (Fig. S4). We observed a mean 9.91 log VLPs g^−1^ (ranging between 8.44 and 10.34 log VLPs g^−1^) of DNA viruses in all samples. Interestingly, we observed significantly more VLPs in the diarrheic samples than in the normal samples (Mann-Whitney *U* test, *P < *0.05; Fig. 6B). The rectal luminal transcription data combined with bacterial community analysis and viral enumeration collectively suggest that the gut inflammation induced by diarrhea may increase the frequency of prophage induction of gut bacteriophages and, possibly, microbial horizontal gene transfer within a specific bacterial group, such as the family *Enterobacteriaceae*.

10.1128/mSystems.00816-20.4 FIG S4 Virus-like particles (VLPs) from the normal and diarrheic samples. Filtrates of the rectal luminal content from the normal and diarrheic calves were stained with SYBR gold. VLPs were then visualized under an epi-fluorescence microscope. The SM buffer filtered through a 0.02-μm pore size filter was used as a negative control. Download FIG S4, JPG file, 0.2 MB. Copyright © 2021 Whon et al. 2021 Whon et al. https://creativecommons.org/licenses/by/4.0/ This content is distributed under the terms of the Creative Commons Attribution 4.0 International license.

### Administration of calf nontoxigenic *Enterobacteriaceae* leads to the diarrheic symptoms in preweaning mice.

**FIG 7:**
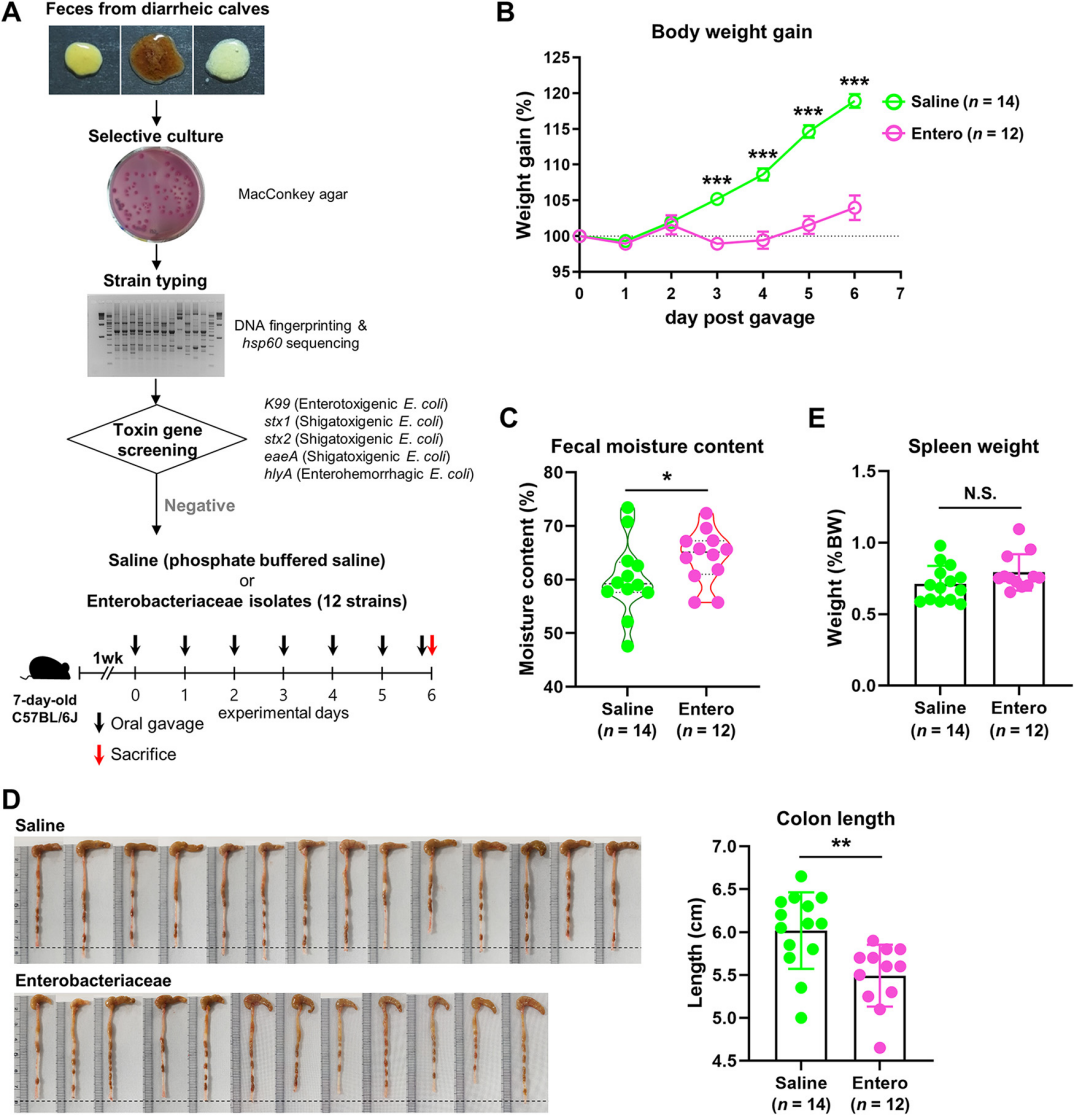
An assessment of the diarrheic symptoms of preweaning mice in response to feeding calf nontoxigenic *Enterobacteriaceae*. (A) Schematic design for the mouse *Enterobacteriaceae* feeding experiments. (B) Body weight gain of the mice fed the nontoxigenic *Enterobacteriaceae* mixture (Entero, *n *= 12) and phosphate-buffered saline (Saline, *n *= 14). (C) Fecal moisture content at day 6 post gavage. (D and E) Images of colon and colon length (D) and spleen weight (E) were obtained after sacrifice. The body weight gain data are presented as a percentage of the initial body weight. The spleen weight data are presented as a percentage of the body weight. The data were analyzed by using the unpaired Student’s *t* test (two-tailed, panels B, D, and E) and Chi-square test (panel C). Data are shown as mean ± SEM. *, *P* < 0.05; **, *P* < 0.01; ***, *P* < 0.001.

We next evaluated the causative role of the alteration of the gut microbiota (i.e., the increased abundance of the family *Enterobacteriaceae*) in the diarrheic symptoms in preweaning animals. To this end, we isolated 12 nontoxigenic *Enterobacteriaceae* members from the rectal luminal samples of the diarrheic calves. A list of isolates and a DNA fingerprint gel image of enterobacterial repetitive intergenic consensus (ERIC) PCR products are provided in Fig. S5. We then treated a mixture of the strain cultures by oral gavage to preweaning mice for 6 continuous days (Entero, *n *= 12; Fig. 7A). Age-matched mice gavaged with phosphate-buffered saline (PBS) were included as a control (Saline, *n *= 14). Feeding the nontoxigenic *Enterobacteriaceae* mixture resulted in a significantly decreased body weight gain in mice after day 3 postgavage (unpaired Student's *t* test, *P < *0.001; Fig. 7B). Fecal moisture content at day 6 postgavage showed a significantly higher level in the Entero group than in the Saline group (Chi-square test, *P = *0.018; Fig. 7C). We additionally observed a significantly shortened colon length in the Entero group compared to that of the Saline group (unpaired Student’s *t* test, *P < *0.01; Fig. 7D), whereas no difference was found in spleen weight between the groups (Fig. 7E). Collectively, the above-described results suggested that the increased abundance of nontoxigenic *Enterobacteriaceae* is capable of causing the gut environment to be diarrheagenic, without systemic inflammation in preweaning animals, including cattle and mouse.

10.1128/mSystems.00816-20.5 FIG S5 *Enterobacteriaceae* strain isolation from diarrheic calves. (A) List of bacterial isolates belonging to the family *Enterobacteriaceae*. (B) Band patterns of ERIC PCR product. Download FIG S5, JPG file, 0.8 MB. Copyright © 2021 Whon et al. 2021 Whon et al. https://creativecommons.org/licenses/by/4.0/ This content is distributed under the terms of the Creative Commons Attribution 4.0 International license.

## DISCUSSION

In the gut environment, the abundant microbial taxa are expected to play important roles during the onset and/or exacerbation of intestinal diseases. However, because the DNA abundance and RNA abundance (gene expression) of gut microbes are not always concordant, recent evidence suggests that a dominant transcribing organism is more capable of exerting an effect on disease severity than a numerically dominant organism (34). In this context, one can postulate that the dysbiosis of the gut microbiome during the progression of intestinal diseases can be subdivided into a microbial compositional dysbiosis and functional dysbiosis, both of which may be individually characterized.

In the current study, we performed 16S rRNA gene-based metataxonomic analysis combined with untargeted transcriptional profiling to gain insight into the dysbiotic signature of the gut microbiome that accompanies diarrhea in calves. In terms of the microbial compositional dysbiosis, the presented data highlight cases in which the abundance of the family *Enterobacteriaceae* is elevated in the diarrheic gut. Together with this compositional change signature, the data revealed a positive correlation between the temporal compositional changes in the abundance of *Enterobacteriaceae* and the diarrheic severity (i.e., the Bristol stool scale). In addition, the data reflected microbial functional dysbiosis, as evidenced by elevated aerobic respiration and virulence potential in the diarrheic microbiome. It is also worth mentioning that, based on the metataxonomic analysis, the family *Enterobacteriaceae* was not the most abundant taxon in the gut of diarrheic calves, but the total transcriptional characteristic of the diarrheic microbiome was considerably affected by this single family (Fig. S2 and S3).

By conducting both the diagnostic PCR and metataxonomic analyses, we attempted to determine the origin of the *Proteobacteria* taxa in the normal and diarrheic samples and concluded that most of them represented autochthonous microbiota. Indeed, several studies published in recent years reported the presence of the *Proteobacteria* species in the gut of mammalian infants, including human (35), mouse (36), pig (37), and the giant panda (38). As recently reviewed by us, these bacteria (transmitted probably from the mother) play important roles in preparing the neonatal gut for the successive colonization of late colonizers, e.g., strict anaerobes (7). Nonetheless, the results presented here do not support the notion that the known allochthonous etiological agents are irrelevant to the onset of diarrhea or that the increase in the abundance of *Enterobacteriaceae* is the direct cause of calf diarrhea. Our study is also limited because we could not confirm whether the *Proteobacteria* observed in the gut of diarrheic calves are autochthonous (i.e., colonizers of the gut) or allochthonous (i.e., merely passing through after environmental exposure). Nevertheless, the current study highlights the delicate intestinal state that is prone to increased *Proteobacteria* abundance and furthers the risk of diarrhea development in preweaning calves.

Based on the host transcriptomics data, the elevated activities of sulfide oxidases (as evidenced by the expression data with enriched expression of genes related to sulfide oxidation to sulfate and molybdenum cofactor biosynthesis) and arylsulfatases indicated elevated sulfate levels in the gut of diarrheic calves. The increased level of bacterial cytochrome *c* activity in the diarrheic gut supported an elevated abundance of sulfate rather than sulfide, which inhibits the cytochrome *c*-dependent aerobic respiration (39). The early (e.g., E. coli) and late colonizers (e.g., strictly anaerobic sulfate-reducing bacteria) are capable of using sulfate in assimilatory (e.g., reducing sulfate to synthesize sulfur-containing cell components) and dissimilatory sulfate reduction (e.g., using sulfate as a terminal electron acceptor to obtain energy), respectively (40, 41). However, the latter is not likely to occur in the diarrheic gut, where robust bacterial aerobic respiration takes place. In E. coli, the inner membrane protein CysZ mediates the import of sulfate for assimilatory reduction, and intriguingly, this energetically unfavorable process is regulated by the extracellular pH (42). Under acidic conditions, sulfate ions cross the periplasmic membrane together with cations, thereby temporarily neutralizing the negative charge of the membrane. Indeed, breastfeeding leads to an acidic environment in the infant intestine (43). Collectively, the results of the current study imply the existence of a “prolonged symbiotic relationship” between the preweaning diarrheic calf and the autochthonous gut *Enterobacteriaceae*. The host gut provides sulfate to enable these microbes to dominate and takes advantage of the microbial aerobic respiration until the gut becomes hypoxic.

Nevertheless, it appears that the prolonged symbiotic relationship exposes the calf gut to several immune challenges. As shown in Fig. 5, elevated NF-κB-dependent innate immune responses are among the most important features of the transcriptomes of diarrheic calves. Considering that the *Enterobacteriaceae* that were abundant in the diarrheic gut showed enhanced transcriptional activity for the biosynthesis of UDP-GlcNAc (Fig. 4), elevated amounts of antigenic cell wall components liberated from these microbial cells might play important roles in inducing excessive immune responses in the host calf. Importantly, the *Enterobacteriaceae*-mediated excessive immune responses in the gut of diarrheic calves are reminiscent of the *Proteobacteria*-mediated gut inflammation in monogastric animals. Under these conditions, autochthonous *Proteobacteria* (e.g., E. coli) impose selective forces that confer a fitness advantage upon the closely related bacterial species (e.g., S. enterica and Campylobacter jejuni), rendering the host gut more susceptible to infection with allochthonous enteric pathogens (44, 45). The mechanisms underlying this “like will to like” concept were, indeed, characterized. An expansion of *Proteobacteria* results in both increased gut inflammation and decreased abundance of butyrate-producing bacteria, promoting infection with gut pathogens via the bacterial molybdenum cofactor-dependent metabolic pathways (46) and host-provided lactate (47).

While the untargeted transcriptomics analysis of the rectal luminal content enabled profiling of the normal and diarrhea-associated gene expression in the bovine host in the current study, the relatively small number of the host RNA reads (a mean of 179-,134 reads for samples with over 0.05% [>20,000 reads] mapping rate) limited a thorough investigation of the bovine host transcriptome. Future transcriptomics analysis of the host intestinal tissues may enable more robust disease-associated host transcriptome profiling in calves. Collectively, our data suggest that the increased incidence of diarrheic symptoms in young calves is attributed primarily to an imbalance in the gut microbiota. In this context, approaches that aggravate gut dysbiosis (e.g., antibiotic treatment) might not be an effective treatment for calf diarrhea; indeed, such treatment induced recurrence of diarrhea in many cases. Rather, an approach that balances the gut microbiota under dysbiotic conditions (i.e., transplantation of fecal microbiota from a healthy donor to a diarrheic recipient) will be of interest to ameliorate calf diarrhea.

## MATERIALS AND METHODS

### Sample collection.

The study protocol was approved by the Institutional Review Boards of the Kyung Hee University [KHUASP(SE)-17-027]. Calves (including their mothers) treated with antibiotics or other medications within a month of sampling were excluded from further analysis. Rectal material was sampled from 111 Hanwoo calves as follows: single sample collection from normal (N, *n *= 53) and diarrheic calves (D, *n *= 53) and multiple sample collection from calves with repeated normal diarrhea (ND, *n *= 5) (Table S1). For the diarrheic and ND groups, the samples were collected from calves exhibiting initial or acute diarrheic symptoms under daily observation. In the ND group, calves exhibiting diarrheic symptoms for 2 continuous days were given an electrolyte solution to prevent dehydration. The mothers nurtured their calves in individual barns. Calves that exhibited severe diarrheic symptoms were isolated from their mothers for 4 to 5 h to avoid milk feeding. Otherwise, the calves moved in and out of their mother’s cage through a calf passage. To obtain samples from the calf environment, we additionally sampled the feces, feed pellet, water, bedding, and maternal milk and feces of normal (*n *= 6) and diarrheic calves (*n *= 5). Rectal luminal content was collected by rectal enema using clean disposable latex gloves. The experiments were performed in agreement with the ARRIVE guidelines (48). The collected samples were transported to the laboratory on dry ice and stored at −80°C until use.

### RNA extraction and RNA-Seq.

RNA was isolated from the calf rectal luminal content using TRIzol reagent (Life Technologies, Carlsbad, CA, USA). cDNA library preparation was conducted using the TruSeq stranded total RNA low-throughput (LT) sample prep kit (Ribo-Zero human/mouse/rat) according to the manufacturer’s instructions (Illumina, San Diego, CA, USA). cDNA sequencing of 18 samples (*n *= 9 each for the normal and diarrheic groups) was performed with an Illumina HiSeq 4000 instrument in 151-paired-end mode. The raw reads were quality-filtered using Trimmomatic (v0.36) software (49) and processed for further rectal transcriptome data analysis.

### Bovine host transcriptomics analysis.

*B. taurus* reference genome (Bos_taurus.UMD3.1.dna_toplevel.fa) and gene transfer format (Bos_taurus.UMD3.1.89.gtf) files were downloaded from the Ensembl website (https://asia.ensembl.org/index.html). Quality-filtered paired-end reads were aligned with the *B. taurus* reference genome using the TopHat2 aligner (50). The mapped reads were assembled, merged, and visualized according to the Tuxedo protocol (51), which includes the Cufflinks, Cuffmerge, Cuffdiff, and CummeRbund packages from R. A density plot was generated from Cuffdiff-calculated log_10_ fragments per kilobase million (FPKM) values. For pathway abundance analysis, the mapped calf rectal transcripts (accepted_hit.bam generated by TopHat2) were normalized using DESeq2 (52) and subsequently annotated using the Reactome Pathway Knowledgebase (53).

**Bacterial metatranscriptomics analysis.** The quality-filtered paired-end reads were functionally profiled using the HUMAnN2 software package (54), according to the HUMAnN2 user manual (https://huttenhower.sph.harvard.edu/humann2). Briefly, the reads were mapped to the sample-specific pangenomes using Bowtie2 (55), and the subsequent unmapped reads were mapped to UniRef90 (56) using DIAMOND translated search (57). The assigned reads were counted per gene family and normalized based on their length and alignment quality. Gene family abundances were then combined into structured pathways from MetaCyc (26) and sum-normalized to the relative abundances. The HUMAnN2 output files (gene family and pathway abundance files) were used as input files to perform the core diversity analysis using the QIIME software package (v1.9.0) (58).

**Viral RNA analysis.** rRNA reads were subtracted from the quality-filtered paired-end reads by assigning them to the SILVA SSU (16S/18S) and LSU (23S/28S) databases using the SortMeRNA (v2.1) software (59). The resulting non-rRNA reads were subjected to a blastn search against the RefSeq viral genome database. The sequence identity and query coverage were set to 95% and 90%, respectively, for high sensitivity and low false-positive rate.

### DNA extraction and Illumina sequencing of bacterial 16S rRNA genes.

Bacterial genomic DNA was extracted from the calf rectal luminal content (129 samples from 111 calves, see Table S1) using repeated bead beating and a column method (60). A fragment of the 16S rRNA gene spanning the hypervariable V3-V4 regions was amplified by PCR using the forward primer 5′-TCG TCG GCA GCG TCA GAT GTG TAT AAG AGA CAG CCT ACG GGN GGC WGC AG-3′ and the reverse primer 5′-GTC TCG TGG GCT CGG AGA TGT GTA TAA GAG ACA GGA CTA CHV GGG TAT CTA ATC C-3′. PCR was performed in a C 1000 thermal cycler (Bio-Rad, Hercules, CA, USA). The PCR conditions were as follows: initial denaturation at 95°C for 3 min followed by 23 cycles of denaturation at 95°C for 30 s, annealing at 55°C for 30 s, and extension at 72°C for 30 s. A final extension step was performed at 72°C for 5 min. Products of three PCRs with the same template were pooled. We investigated the possible DNA contamination of all reagents used for DNA extraction. PCR analysis targeting the hypervariable V3-V4 regions of the 16S rRNA gene (30-cycle reaction) revealed no apparent contamination of any reagents used (Fig. S6A). PCR amplicons from DNA extracted from the ZymoBIOMICS microbial community standard (ZYMO Research) (*n *= 2) and “blank” negative DNA extraction/PCR controls (i.e., PCR products of template acquired from a sham extraction to which no rectal luminal sample was added; *n *= 2) were included as mock community (positive) and negative controls for the bacterial 16S rRNA gene analysis, respectively. The 16S V3-4 PCR product library was prepared using the Nextera XT index (Illumina). The library was sequenced on an Illumina MiSeq platform using the paired-end 2 × 300-bp reagent kit according to the manufacturer’s instructions.

10.1128/mSystems.00816-20.6 FIG S6 The positive and negative controls for the bacterial 16S rRNA gene sequence analysis. (A) The possible DNA contamination of all reagents used for DNA extraction was tested by PCR analysis targeting the hypervariable V3-V4 regions of the 16S rRNA gene (30-cycle reaction). DNA extracted from ZymoBIOMICS microbial community standard was used as a positive PCR control. (B) Overview of the 16S rRNA data set for the positive and negative controls generated using MiSeq. *, PCR amplicons from DNA extracted from ZymoBIOMICS microbial community standard (ZYMO Research); #, “blank” negative DNA extraction/PCR controls (i.e., PCR products of template acquired from a sham extraction to which no rectal luminal sample is added). (C) Relative abundance of the bacterial 16S rRNA sequences in the mock community standard (*n *= 2). The taxon names in parentheses correspond to the taxonomic annotation in the current study. Download FIG S6, JPG file, 0.6 MB. Copyright © 2021 Whon et al. 2021 Whon et al. https://creativecommons.org/licenses/by/4.0/ This content is distributed under the terms of the Creative Commons Attribution 4.0 International license.

### Bacterial 16S rRNA gene sequence analysis.

The adapter sequences were trimmed from the raw fastq files, and the trimmed reads were demultiplexed according to the samples using the bcl2fastq2 conversion software v2.20.0. (Illumina). The sorted reads were imported and processed using QIIME2 v2018.11 (61) for further bioinformatics analyses. The imported paired reads were quality filtered, denoised, and merged using the plugin DADA2 (62) to generate the ASV feature table. Chimeric sequences and singleton ASVs were excluded from further analyses. Taxonomic classification was performed using the plugin q2-feature-classifier using the classify-sklearn method (63) and the pretrained SILVA v132 database (64) with 99% identity. To determine the species diversity in each sample, alpha and beta diversity analyses were performed using the plugin q2-diversity in QIIME2 v2018.11 at a sampling depth of 21,542 reads for normal and diarrheic calves, and 28,018 reads for intermittently diarrheic calves. The 16S rRNA data set generated using MiSeq for the positive and negative controls is summarized in Fig. S6B. For taxonomic annotation of the mock community standard (positive control), a representative sequence for each operational taxonomic unit (OTU) was aligned with the sequences in the SILVA 123 QIIME-compatible database using the QIIME software. The taxonomic annotation data for the positive and negative controls are shown in Fig. S6C and Table S3, respectively. The majority of the assigned reads in the negative control were highly unlikely to be present in the calf rectal luminal content, suggesting no (or very little) impact of contamination on the 16S rRNA gene analysis.

10.1128/mSystems.00816-20.9 TABLE S3 Taxonomic annotation data for the negative control. Download Table S3, PDF file, 0.2 MB. Copyright © 2021 Whon et al. 2021 Whon et al. https://creativecommons.org/licenses/by/4.0/ This content is distributed under the terms of the Creative Commons Attribution 4.0 International license.

### Diagnostic multiplex PCR assay.

To detect the presence of RNA viruses and bacterial virulence genes, RNA and DNA, respectively, were isolated from the calf rectal luminal content. The isolation methods were as described above. A multiplex PCR assay was performed using previously published primer sequences (Table 1). The PCR conditions for the detection of RNA viruses were as follows: initial denaturation at 94°C for 2 min followed by 30 cycles of denaturation at 94°C for 30 s, annealing at 60°C for 30 s, and extension at 72°C for 20 s. The PCR conditions for the detection of bacterial virulence genes were as follows: initial denaturation at 94°C for 2 min followed by 30 cycles of denaturation at 94°C for 40 s, annealing at 62°C for 50 s, and extension at 72°C for 50 s. A final extension step at 72°C for 5 min was performed for both reaction types.

### Enumeration of rectal luminal VLPs.

VLPs were enumerated as described previously (17). Briefly, a 0.1-g sample of the rectal luminal content was suspended in 10 ml of sterilized saline magnesium buffer (SM buffer; 100 mM NaCl, 8 mM MgSO_4_, 50 mM Tris-HCl [pH 7.4], and 0.002% gelatin; filtered through a 0.02-μm Anodisc polycarbonate filter [Whatman] before use). After serial filtration through 5-, 0.45-, and 0.2-μm pore size syringe filters (Sartorius), the filtrate was serially diluted 10-fold, and the same dilutions of the normal and diarrheic samples were compared. The filtrates were then filtered through a 0.02-μm Anodisc filter. The filters were stained with 5× SYBR gold for DNA viruses for 10 min, washed once, and visualized under an Eclipse 50*i* microscope (Nikon) equipped with an Intensilight C-HGFI device (Nikon). VLP images (×1,000 magnification) were obtained, 10 images from different fields of view per sample, and VLPs were counted using an *i*-Solution image analyzer (InnerView, Seoul, South Korea). The SM buffer was used as a negative control.

### Real-time quantitative PCR.

To determine the abundance of several toxin genes of pathogenic E. coli (i.e., Shiga toxin type 2 [*stx*_2_], enterohemorrhagic E. coli O157:H7-specific intimin [*eaeA*], and plasmid-encoded enterohemolysin [*hlyA*]), DNA was prepared from the calf rectal luminal content as described above. Samples were analyzed in 9 biological and 2 technical replicates. The primer sets are listed in Table 1. The bacterial 16S rRNA gene (primers Bac1055YF and Bac1392R) was used as the control (65). PCR was performed in a reaction volume of 25 μl, containing 12.5 μl of SYBR premix *Ex Taq* (TaKaRa, Shiga, Japan), 10 pmol each of the forward and reverse primers, and 2 μl of template DNA (<25 ng), using a CFX96 real-time PCR detection system (Bio-Rad, Hercules, CA, USA). The values are presented as the relative amount.

### Moisture content analysis.

The moisture content in calf rectal samples was determined in two technical replicates of 0.1 g of frozen homogenized rectal material (−80°C) as the percentage of mass loss after lyophilization.

### Nontoxigenic *Enterobacteriaceae* strain preparation.

For isolation of indigenous *Enterobacteriaceae* strains in the diarrheic calves, the rectal luminal content samples from six diarrheic calves were suspended in sterile PBS and serially diluted in 10-fold steps, and 10^−4^ to 10^−6^ diluents were spread onto MacConkey agar medium. The agar plates were incubated at 37°C under ambient aerobic or anaerobic conditions in an anaerobic chamber (Bactron II-2, Sheldon Manufacturing, Oregon, USA) filled with 5% H_2_, 5% CO_2_, and 90% N_2_ atmosphere. After 48 h of incubation, 31 randomly selected colonies were purified by repeated transfer and subjected to species identification (16S rRNA gene sequencing) and strain typing (partial *hsp60* gene sequencing and enterobacterial repetitive intergenic consensus [ERIC] PCR [66]). Excluding the strains positive for toxin gene (*K99*, *LT1*, *LT2*, *ST1*, *ST2*, *stx*_1_, *stx*_2_, *eaeA*, and *hlyA*) PCR or duplicated strains, 12 *Enterobacteriaceae* strains were administered to preweaning mice as a mixture. Overnight pure cultures of each strain, grown on Luria-Bertani agar medium, were harvested, suspended in sterile PBS, and washed twice by vortex and centrifugation at 11,000 × *g* for 10 min. The pellets were resuspended and pooled to 2 × 10^9^ CFU/ml in PBS.

### Mice.

Seven-day-old C57BL/6J mice from 6 dams kept under specific-pathogen-free conditions were purchased from CLS Bio (Bucheon, Republic of Korea) and housed in individually ventilated cages with sterilized bedding. Littermates were cohoused with their dams during experiments. Mice were supplied with autoclaved water and a sterilized normal-chow diet *ad libitum*. After 1 week of acclimatization, 6 cages were randomly assigned to control (3 cages, 14 pups) or experimental groups (3 cages, 12 pups). The pups were orally administered with 100 μl of PBS (for the Saline group) or Enterobacteriaceae culture suspension (for the Entero group) daily for 6 days.

### Statistics.

The statistical analyses were performed using Prism v8.1.2 for Windows (GraphPad Software, La Jolla, CA, USA). Comparisons between two samples were made using the nonparametric Mann-Whitney *U* test (one-tailed) for the cattle study and unpaired Student's *t* test (two-tailed) for the mouse study. After LEfSe analysis, the corresponding data were reanalyzed using a multiple *t* test. Corrections for multiple comparisons were made using the false-discovery rate (FDR; threshold of 0.05). Comparisons of fecal moisture content were conducted using the Chi-square test. Comparisons between multiple samples were conducted with the analysis of variance (ANOVA), followed by Tukey’s *post hoc* test. (*, *P* < 0.05; **, *P* < 0.01; ***, *P* < 0.001). The statistical significance for observed variations was assessed using the function “PERMANOVA” with 999 permutations. The lines, boxes, and whiskers in the box-plot diagrams represent the median, first and third quartiles, and min-to-max distribution of replicate values, respectively. The values and scattered dots in the bar graphs represent the means ± standard error of the mean (SEM) and the individual replicates, respectively.

### Data availability.

The sequences of the 16S rRNA genes and cDNA obtained from the rectal luminal content of calves have been deposited in the European Nucleotide Archive and are available under the accession number PRJEB25741.

## References

[1] Cho YI, Yoon KJ. 2014. An overview of calf diarrhea: infectious etiology, diagnosis, and intervention. J Vet Sci 15:1–17. doi:10.4142/jvs.2014.15.1.1. 24378583 PMC3973752

[2] USDA. 2012. Dairy heifer raiser, 2011. USDA-APHIS-VS, CEAH, National Animal Health Monitoring System (NAHMS), Fort Collins, CO. https://www.aphis.usda.gov/animal_health/nahms/dairy/downloads/dairyheifer11/HeiferRaiser_1.pdf.

[3] Bartels CJ, Holzhauer M, Jorritsma R, Swart WA, Lam TJ. 2010. Prevalence, prediction and risk factors of enteropathogens in normal and non-normal faeces of young Dutch dairy calves. Prev Vet Med 93:162–169. doi:10.1016/j.prevetmed.2009.09.020. 19819574 PMC7125667

[4] Tsuchiaka S, Masuda T, Sugimura S, Kobayashi S, Komatsu N, Nagai M, Omatsu T, Furuya T, Oba M, Katayama Y, Kanda S, Yokoyama T, Mizutani T. 2016. Development of a novel detection system for microbes from bovine diarrhea by real-time PCR. J Vet Med Sci 78:383–389. doi:10.1292/jvms.15-0552. 26616156 PMC4829504

[5] Cho YI, Han JI, Wang C, Cooper V, Schwartz K, Engelken T, Yoon KJ. 2013. Case-control study of microbiological etiology associated with calf diarrhea. Vet Microbiol 166:375–385. doi:10.1016/j.vetmic.2013.07.001. 23886509 PMC7117237

[6] Mackie RI, Sghir A, Gaskins HR. 1999. Developmental microbial ecology of the neonatal gastrointestinal tract. Am J Clin Nutr 69:1035S–1045S. doi:10.1093/ajcn/69.5.1035s. 10232646

[7] Shin NR, Whon TW, Bae JW. 2015. Proteobacteria: microbial signature of dysbiosis in gut microbiota. Trends Biotechnol 33:496–503. doi:10.1016/j.tibtech.2015.06.011. 26210164

[8] Koren O, Goodrich JK, Cullender TC, Spor A, Laitinen K, Backhed HK, Gonzalez A, Werner JJ, Angenent LT, Knight R, Backhed F, Isolauri E, Salminen S, Ley RE. 2012. Host remodeling of the gut microbiome and metabolic changes during pregnancy. Cell 150:470–480. doi:10.1016/j.cell.2012.07.008. 22863002 PMC3505857

[9] Guaraldi F, Salvatori G. 2012. Effect of breast and formula feeding on gut microbiota shaping in newborns. Front Cell Infect Microbiol 2:94. doi:10.3389/fcimb.2012.00094. 23087909 PMC3472256

[10] Morrow AL, Lagomarcino AJ, Schibler KR, Taft DH, Yu Z, Wang B, Altaye M, Wagner M, Gevers D, Ward DV, Kennedy MA, Huttenhower C, Newburg DS. 2013. Early microbial and metabolomic signatures predict later onset of necrotizing enterocolitis in preterm infants. Microbiome 1:13. doi:10.1186/2049-2618-1-13. 24450576 PMC3971624

[11] Yang WH, Heithoff DM, Aziz PV, Sperandio M, Nizet V, Mahan MJ, Marth JD. 2017. Recurrent infection progressively disables host protection against intestinal inflammation. Science 358:eaao5610. doi:10.1126/science.aao5610. 29269445 PMC5824721

[12] Sommer F, Backhed F. 2013. The gut microbiota: masters of host development and physiology. Nat Rev Microbiol 11:227–238. doi:10.1038/nrmicro2974. 23435359

[13] Wotzka SY, Nguyen BD, Hardt WD. 2017. Salmonella Typhimurium diarrhea reveals basic principles of enteropathogen infection and disease-promoted DNA exchange. Cell Host Microbe 21:443–454. doi:10.1016/j.chom.2017.03.009. 28407482

[14] Mirzaei MK, Maurice CF. 2017. Menage à trois in the human gut: interactions between host, bacteria and phages. Nat Rev Microbiol 15:397–408. doi:10.1038/nrmicro.2017.30. 28461690

[15] Kim MS, Bae JW. 2016. Spatial disturbances in altered mucosal and luminal gut viromes of diet-induced obese mice. Environ Microbiol 18:1498–1510. doi:10.1111/1462-2920.13182. 26690305

[16] Norman JM, Handley SA, Baldridge MT, Droit L, Liu CY, Keller BC, Kambal A, Monaco CL, Zhao G, Fleshner P, Stappenbeck TS, McGovern DP, Keshavarzian A, Mutlu EA, Sauk J, Gevers D, Xavier RJ, Wang D, Parkes M, Virgin HW. 2015. Disease-specific alterations in the enteric virome in inflammatory bowel disease. Cell 160:447–460. doi:10.1016/j.cell.2015.01.002. 25619688 PMC4312520

[17] Yang JY, Kim MS, Kim E, Cheon JH, Lee YS, Kim Y, Lee SH, Seo SU, Shin SH, Choi SS, Kim B, Chang SY, Ko HJ, Bae JW, Kweon MN. 2016. Enteric viruses ameliorate gut inflammation via Toll-like receptor 3 and Toll-like receptor 7-mediated interferon-beta production. Immunity 44:889–900. doi:10.1016/j.immuni.2016.03.009. 27084119

[18] Kim MS, Bae JW. 2018. Lysogeny is prevalent and widely distributed in the murine gut microbiota. ISME J 12:1127–1141. doi:10.1038/s41396-018-0061-9. 29416123 PMC5864201

[19] Diard M, Bakkeren E, Cornuault JK, Moor K, Hausmann A, Sellin ME, Loverdo C, Aertsen A, Ackermann M, De Paepe M, Slack E, Hardt WD. 2017. Inflammation boosts bacteriophage transfer between Salmonella spp. Science 355:1211–1215. doi:10.1126/science.aaf8451. 28302859

[20] Talley NJ, Weaver AL, Zinsmeister AR, Melton LJ. 1994. Self-reported diarrhea: what does it mean. Am J Gastroenterol 89:1160–1164. 8053428

[21] Lewis SJ, Heaton KW. 1997. Stool form scale as a useful guide to intestinal transit time. Scand J Gastroenterol 32:920–924. doi:10.3109/00365529709011203. 9299672

[22] Segata N, Izard J, Waldron L, Gevers D, Miropolsky L, Garrett WS, Huttenhower C. 2011. Metagenomic biomarker discovery and explanation. Genome Biol 12:R60. doi:10.1186/gb-2011-12-6-r60. 21702898 PMC3218848

[23] Thompson LR, Sanders JG, McDonald D, Amir A, Ladau J, Locey KJ, Prill RJ, Tripathi A, Gibbons SM, Ackermann G, Navas-Molina JA, Janssen S, Kopylova E, Vazquez-Baeza Y, Gonzalez A, Morton JT, Mirarab S, Zech Xu Z, Jiang L, Haroon MF, Kanbar J, Zhu Q, Jin Song S, Kosciolek T, Bokulich NA, Lefler J, Brislawn CJ, Humphrey G, Owens SM, Hampton-Marcell J, Berg-Lyons D, McKenzie V, Fierer N, Fuhrman JA, Clauset A, Stevens RL, Shade A, Pollard KS, Goodwin KD, Jansson JK, Gilbert JA, Knight R, Earth Microbiome Project Consortium. 2017. A communal catalogue reveals Earth’s multiscale microbial diversity. Nature 551:457–463. doi:10.1038/nature24621. 29088705 PMC6192678

[24] Ley RE, Lozupone CA, Hamady M, Knight R, Gordon JI. 2008. Worlds within worlds: evolution of the vertebrate gut microbiota. Nat Rev Microbiol 6:776–788. doi:10.1038/nrmicro1978. 18794915 PMC2664199

[25] Bakdash JZ, Marusich LR. 2017. Repeated measures correlation. Front Psychol 8:456. doi:10.3389/fpsyg.2017.00456. 28439244 PMC5383908

[26] Caspi R, Billington R, Ferrer L, Foerster H, Fulcher CA, Keseler IM, Kothari A, Krummenacker M, Latendresse M, Mueller LA, Ong Q, Paley S, Subhraveti P, Weaver DS, Karp PD. 2016. The MetaCyc database of metabolic pathways and enzymes and the BioCyc collection of pathway/genome databases. Nucleic Acids Res 44:D471–D480. doi:10.1093/nar/gkv1164. 26527732 PMC4702838

[27] Naseem S, Konopka JB. 2015. N-acetylglucosamine regulates virulence properties in microbial pathogens. PLoS Pathog 11:e1004947. doi:10.1371/journal.ppat.1004947. 26226264 PMC4520607

[28] Shen A, Kamp HD, Grundling A, Higgins DE. 2006. A bifunctional O-GlcNAc transferase governs flagellar motility through anti-repression. Genes Dev 20:3283–3295. doi:10.1101/gad.1492606. 17158746 PMC1686605

[29] Williams JM, Duckworth CA, Burkitt MD, Watson AJ, Campbell BJ, Pritchard DM. 2015. Epithelial cell shedding and barrier function: a matter of life and death at the small intestinal villus tip. Vet Pathol 52:445–455. doi:10.1177/0300985814559404. 25428410 PMC4441880

[30] Stauber J, Shaikh N, Ordiz MI, Tarr PI, Manary MJ. 2016. Droplet digital PCR quantifies host inflammatory transcripts in feces reliably and reproducibly. Cell Immunol 303:43–49. doi:10.1016/j.cellimm.2016.03.007. 27063479 PMC4863679

[31] Mandl P, Kiss JP. 2007. Role of presynaptic nicotinic acetylcholine receptors in the regulation of gastrointestinal motility. Brain Res Bull 72:194–200. doi:10.1016/j.brainresbull.2007.02.005. 17452281

[32] Finkbeiner SR, Allred AF, Tarr PI, Klein EJ, Kirkwood CD, Wang D. 2008. Metagenomic analysis of human diarrhea: viral detection and discovery. PLoS Pathog 4:e1000011. doi:10.1371/journal.ppat.1000011. 18398449 PMC2290972

[33] Kim MS, Park EJ, Roh SW, Bae JW. 2011. Diversity and abundance of single-stranded DNA viruses in human feces. Appl Environ Microbiol 77:8062–8070. doi:10.1128/AEM.06331-11. 21948823 PMC3208976

[34] Schirmer M, Franzosa EA, Lloyd-Price J, McIver LJ, Schwager R, Poon TW, Ananthakrishnan AN, Andrews E, Barron G, Lake K, Prasad M, Sauk J, Stevens B, Wilson RG, Braun J, Denson LA, Kugathasan S, McGovern DPB, Vlamakis H, Xavier RJ, Huttenhower C. 2018. Dynamics of metatranscription in the inflammatory bowel disease gut microbiome. Nat Microbiol 3:337–346. doi:10.1038/s41564-017-0089-z. 29311644 PMC6131705

[35] Kostic AD, Gevers D, Siljander H, Vatanen T, Hyotylainen T, Hamalainen AM, Peet A, Tillmann V, Poho P, Mattila I, Lahdesmaki H, Franzosa EA, Vaarala O, de Goffau M, Harmsen H, Ilonen J, Virtanen SM, Clish CB, Oresic M, Huttenhower C, Knip M, Group DS, Xavier RJ, DIABIMMUNE Study Group. 2015. The dynamics of the human infant gut microbiome in development and in progression toward type 1 diabetes. Cell Host Microbe 17:260–273. doi:10.1016/j.chom.2015.01.001. 25662751 PMC4689191

[36] Mirpuri J, Raetz M, Sturge CR, Wilhelm CL, Benson A, Savani RC, Hooper LV, Yarovinsky F. 2014. Proteobacteria-specific IgA regulates maturation of the intestinal microbiota. Gut Microbes 5:28–39. doi:10.4161/gmic.26489. 24637807 PMC4049932

[37] Frese SA, Parker K, Calvert CC, Mills DA. 2015. Diet shapes the gut microbiome of pigs during nursing and weaning. Microbiome 3:28. doi:10.1186/s40168-015-0091-8. 26167280 PMC4499176

[38] Zhang W, Liu W, Hou R, Zhang L, Schmitz-Esser S, Sun H, Xie J, Zhang Y, Wang C, Li L, Yue B, Huang H, Wang H, Shen F, Zhang Z. 2018. Age-associated microbiome shows the giant panda lives on hemicelluloses, not on cellulose. ISME J 12:1319–1328. doi:10.1038/s41396-018-0051-y. 29391488 PMC5931968

[39] Nicholls P, Marshall DC, Cooper CE, Wilson MT. 2013. Sulfide inhibition of and metabolism by cytochrome c oxidase. Biochem Soc Trans 41:1312–1316. doi:10.1042/BST20130070. 24059525

[40] Rossi E, Motta S, Mauri P, Landini P. 2014. Sulfate assimilation pathway intermediate phosphoadenosine 59-phosphosulfate acts as a signal molecule affecting production of curli fibres in Escherichia coli. Microbiology (Reading) 160:1832–1844. doi:10.1099/mic.0.079699-0. 24934621

[41] Carbonero F, Benefiel AC, Alizadeh-Ghamsari AH, Gaskins HR. 2012. Microbial pathways in colonic sulfur metabolism and links with health and disease. Front Physiol 3:448. doi:10.3389/fphys.2012.00448. 23226130 PMC3508456

[42] Zhang L, Jiang WS, Nan J, Almqvist J, Huang YF. 2014. The Escherichia coli CysZ is a pH dependent sulfate transporter that can be inhibited by sulfite. Biochim Biophys Acta 1838:1809–1816. doi:10.1016/j.bbamem.2014.03.003. 24657232

[43] Walker WA, Iyengar RS. 2015. Breast milk, microbiota, and intestinal immune homeostasis. Pediatr Res 77:220–228. doi:10.1038/pr.2014.160. 25310762

[44] Winter SE, Baumler AJ. 2014. Why related bacterial species bloom simultaneously in the gut: principles underlying the ‘Like will to like’ concept. Cell Microbiol 16:179–184. doi:10.1111/cmi.12245. 24286560 PMC4013256

[45] Stecher B, Chaffron S, Kappeli R, Hapfelmeier S, Freedrich S, Weber TC, Kirundi J, Suar M, McCoy KD, von Mering C, Macpherson AJ, Hardt WD. 2010. Like will to like: abundances of closely related species can predict susceptibility to intestinal colonization by pathogenic and commensal bacteria. PLoS Pathog 6:e1000711. doi:10.1371/journal.ppat.1000711. 20062525 PMC2796170

[46] Zhu W, Winter MG, Byndloss MX, Spiga L, Duerkop BA, Hughes ER, Buttner L, de Lima Romao E, Behrendt CL, Lopez CA, Sifuentes-Dominguez L, Huff-Hardy K, Wilson RP, Gillis CC, Tukel C, Koh AY, Burstein E, Hooper LV, Baumler AJ, Winter SE. 2018. Precision editing of the gut microbiota ameliorates colitis. Nature 553:208–211. doi:10.1038/nature25172. 29323293 PMC5804340

[47] Gillis CC, Hughes ER, Spiga L, Winter MG, Zhu W, Furtado de Carvalho T, Chanin RB, Behrendt CL, Hooper LV, Santos RL, Winter SE. 2018. Dysbiosis-associated change in host metabolism generates lactate to support Salmonella growth. Cell Host Microbe 23:54–64 e6. doi:10.1016/j.chom.2017.11.006. 29276172 PMC5764812

[48] Kilkenny C, Browne WJ, Cuthill IC, Emerson M, Altman DG. 2010. Improving bioscience research reporting: the ARRIVE guidelines for reporting animal research. PLoS Biol 8:e1000412. doi:10.1371/journal.pbio.1000412. 20613859 PMC2893951

[49] Bolger AM, Lohse M, Usadel B. 2014. Trimmomatic: a flexible trimmer for Illumina sequence data. Bioinformatics 30:2114–2120. doi:10.1093/bioinformatics/btu170. 24695404 PMC4103590

[50] Kim D, Pertea G, Trapnell C, Pimentel H, Kelley R, Salzberg SL. 2013. TopHat2: accurate alignment of transcriptomes in the presence of insertions, deletions and gene fusions. Genome Biol 14:R36. doi:10.1186/gb-2013-14-4-r36. 23618408 PMC4053844

[51] Trapnell C, Roberts A, Goff L, Pertea G, Kim D, Kelley DR, Pimentel H, Salzberg SL, Rinn JL, Pachter L. 2012. Differential gene and transcript expression analysis of RNA-seq experiments with TopHat and Cufflinks. Nat Protoc 7:562–578. doi:10.1038/nprot.2012.016. 22383036 PMC3334321

[52] Love MI, Huber W, Anders S. 2014. Moderated estimation of fold change and dispersion for RNA-seq data with DESeq2. Genome Biol 15:550. doi:10.1186/s13059-014-0550-8. 25516281 PMC4302049

[53] Fabregat A, Jupe S, Matthews L, Sidiropoulos K, Gillespie M, Garapati P, Haw R, Jassal B, Korninger F, May B, Milacic M, Roca CD, Rothfels K, Sevilla C, Shamovsky V, Shorser S, Varusai T, Viteri G, Weiser J, Wu G, Stein L, Hermjakob H, D’Eustachio P. 2018. The Reactome Pathway Knowledgebase. Nucleic Acids Res 46:D649–D655. doi:10.1093/nar/gkx1132. 29145629 PMC5753187

[54] Abubucker S, Segata N, Goll J, Schubert AM, Izard J, Cantarel BL, Rodriguez-Mueller B, Zucker J, Thiagarajan M, Henrissat B, White O, Kelley ST, Methe B, Schloss PD, Gevers D, Mitreva M, Huttenhower C. 2012. Metabolic reconstruction for metagenomic data and its application to the human microbiome. PLoS Comput Biol 8:e1002358. doi:10.1371/journal.pcbi.1002358. 22719234 PMC3374609

[55] Langmead B, Salzberg SL. 2012. Fast gapped-read alignment with Bowtie 2. Nat Methods 9:357–359. doi:10.1038/nmeth.1923. 22388286 PMC3322381

[56] Suzek BE, Huang H, McGarvey P, Mazumder R, Wu CH. 2007. UniRef: comprehensive and non-redundant UniProt reference clusters. Bioinformatics 23:1282–1288. doi:10.1093/bioinformatics/btm098. 17379688

[57] Buchfink B, Xie C, Huson DH. 2015. Fast and sensitive protein alignment using DIAMOND. Nat Methods 12:59–60. doi:10.1038/nmeth.3176. 25402007

[58] Caporaso JG, Kuczynski J, Stombaugh J, Bittinger K, Bushman FD, Costello EK, Fierer N, Pena AG, Goodrich JK, Gordon JI, Huttley GA, Kelley ST, Knights D, Koenig JE, Ley RE, Lozupone CA, McDonald D, Muegge BD, Pirrung M, Reeder J, Sevinsky JR, Turnbaugh PJ, Walters WA, Widmann J, Yatsunenko T, Zaneveld J, Knight R. 2010. QIIME allows analysis of high-throughput community sequencing data. Nat Methods 7:335–336. doi:10.1038/nmeth.f.303. 20383131 PMC3156573

[59] Kopylova E, Noe L, Touzet H. 2012. SortMeRNA: fast and accurate filtering of ribosomal RNAs in metatranscriptomic data. Bioinformatics 28:3211–3217. doi:10.1093/bioinformatics/bts611. 23071270

[60] Yu Z, Morrison M. 2004. Improved extraction of PCR-quality community DNA from digesta and fecal samples. Biotechniques 36:808–812. doi:10.2144/04365ST04. 15152600

[61] Bolyen E, Rideout JR, Dillon MR, Bokulich NA, Abnet C, Al-Ghalith GA, Alexander H, Alm EJ, Arumugam M, Asnicar F, Bai Y, Bisanz JE, Bittinger K, Brejnrod A, Brislawn CJ, Brown CT, Callahan BJ, Caraballo-Rodríguez AM, Chase J, Cope E, Da SR, Dorrestein PC, Douglas GM, Durall DM, Duvallet C, Edwardson CF, Ernst M, Estaki M, Fouquier J, Gauglitz JM, Gibson DL, Gonzalez A, Gorlick K, Guo J, Hillmann B, Holmes S, Holste H, Huttenhower C, Huttley G, Janssen S, Jarmusch AK, Jiang L, Kaehler B, Kang KB, Keefe CR, Keim P, Kelley ST, Knights D, Koester I, Kosciolek T, . 2018. QIIME 2: reproducible, interactive, scalable, and extensible microbiome data science. PeerJ Preprints 6:e27295v2. doi:10.7287/peerj.preprints.27295v2. PMC7015180 31341288

[62] Callahan BJ, McMurdie PJ, Rosen MJ, Han AW, Johnson AJ, Holmes SP. 2016. DADA2: high-resolution sample inference from Illumina amplicon data. Nat Methods 13:581–583. doi:10.1038/nmeth.3869. 27214047 PMC4927377

[63] Pedregosa F, Varoquaux G, Gramfort A, Michel V, Thirion B, Grisel O, Blondel M, Prettenhofer P, Weiss R, Dubourg V, Vanderplas J, Passos A, Cournapeau D, Brucher M, Perrot M, Duchesnay E. 2011. Scikit-learn: machine learning in Python. J Mach Learn Res 12:2825–2830.

[64] Quast C, Pruesse E, Yilmaz P, Gerken J, Schweer T, Yarza P, Peplies J, Glockner FO. 2013. The SILVA ribosomal RNA gene database project: improved data processing and Web-based tools. Nucleic Acids Res 41:D590–D596. doi:10.1093/nar/gks1219. 23193283 PMC3531112

[65] Ritalahti KM, Amos BK, Sung Y, Wu Q, Koenigsberg SS, Loffler FE. 2006. Quantitative PCR targeting 16S rRNA and reductive dehalogenase genes simultaneously monitors multiple Dehalococcoides strains. Appl Environ Microbiol 72:2765–2774. doi:10.1128/AEM.72.4.2765-2774.2006. 16597981 PMC1449079

[66] Stumpf AN, Roggenkamp A, Hoffmann H. 2005. Specificity of enterobacterial repetitive intergenic consensus and repetitive extragenic palindromic polymerase chain reaction for the detection of clonality within the Enterobacter cloacae complex. Diagn Microbiol Infect Dis 53:9–16. doi:10.1016/j.diagmicrobio.2005.04.003. 16182074

[67] Chinsangaram J, Akita GY, Castro AE, Osburn BI. 1993. PCR detection of group A bovine rotaviruses in feces. J Vet Diagn Invest 5:516–521. doi:10.1177/104063879300500403. 8286448

[68] Chang KO, Parwani AV, Saif LJ. 1996. The characterization of VP7 (G type) and VP4 (P type) genes of bovine group A rotaviruses from field samples using RT-PCR and RFLP analysis. Arch Virol 141:1727–1739. doi:10.1007/BF01718295. 8893794

[69] Barman P, Ghosh S, Das S, Varghese V, Chaudhuri S, Sarkar S, Krishnan T, Bhattacharya SK, Chakrabarti A, Kobayashi N, Naik TN. 2004. Sequencing and sequence analysis of VP7 and NSP5 genes reveal emergence of a new genotype of bovine group B rotaviruses in India. J Clin Microbiol 42:2816–2818. doi:10.1128/JCM.42.6.2816-2818.2004. 15184480 PMC427839

[70] Park SJ, Jeong C, Yoon SS, Choy HE, Saif LJ, Park SH, Kim YJ, Jeong JH, Park SI, Kim HH, Lee BJ, Cho HS, Kim SK, Kang MI, Cho KO. 2006. Detection and characterization of bovine coronaviruses in fecal specimens of adult cattle with diarrhea during the warmer seasons. J Clin Microbiol 44:3178–3188. doi:10.1128/JCM.02667-05. 16954245 PMC1594715

[71] Cho KO, Hasoksuz M, Nielsen PR, Chang KO, Lathrop S, Saif LJ. 2001. Cross-protection studies between respiratory and calf diarrhea and winter dysentery coronavirus strains in calves and RT-PCR and nested PCR for their detection. Arch Virol 146:2401–2419. doi:10.1007/s007050170011. 11811688 PMC7087283

[72] Duckmanton L, Carman S, Nagy E, Petric M. 1998. Detection of bovine torovirus in fecal specimens of calves with diarrhea from Ontario farms. J Clin Microbiol 36:1266–1270. doi:10.1128/JCM.36.5.1266-1270.1998. 9574689 PMC104812

[73] Smiley JR, Hoet AE, Traven M, Tsunemitsu H, Saif LJ. 2003. Reverse transcription-PCR assays for detection of bovine enteric caliciviruses (BEC) and analysis of the genetic relationships among BEC and human caliciviruses. J Clin Microbiol 41:3089–3099. doi:10.1128/jcm.41.7.3089-3099.2003. 12843048 PMC165218

[74] Alkan F, Karayel I, Catella C, Bodnar L, Lanave G, Banyai K, Di Martino B, Decaro N, Buonavoglia C, Martella V. 2015. Identification of a bovine enteric calicivirus, Kirklareli virus, distantly related to neboviruses, in calves with enteritis in Turkey. J Clin Microbiol 53:3614–3617. doi:10.1128/JCM.01736-15. 26292294 PMC4609679

[75] Schmitt BJ, Lopez OJ, Ridpath JF, Galeota-Wheeler J, Osorio FA. 1994. Evaluation of PCR for diagnosis of bovine viral diarrhea virus in tissue homogenates. J Vet Diagn Invest 6:44–47. doi:10.1177/104063879400600109. 8011781

[76] Givens MD, Heath AM, Carson RL, Brock KV, Edens MS, Wenzel JG, Stringfellow DA. 2003. Analytical sensitivity of assays used for detection of bovine viral diarrhea virus in semen samples from the southeastern United States. Vet Microbiol 96:145–155. doi:10.1016/S0378-1135(03)00213-X. 14519332

[77] Wu J, Zhang W, Xie B, Wu M, Tong X, Kalpoe J, Zhang D. 2009. Detection and toxin typing of Clostridium perfringens in formalin-fixed, paraffin-embedded tissue samples by PCR. J Clin Microbiol 47:807–810. doi:10.1128/JCM.01324-08. 19109478 PMC2650962

[78] Wang SJ, Yeh DB, Wei CI. 2009. Specific PCR primers for the identification of Salmonella enterica serovar Enteritidis in chicken-related samples. J Food Drug Anal 17:183–189. doi:10.38212/2224-6614.2612.

[79] Lim YH, Hirose K, Izumiya H, Arakawa E, Takahashi H, Terajima J, Itoh K, Tamura K, Kim SI, Watanabe H. 2003. Multiplex polymerase chain reaction assay for selective detection of Salmonella enterica serovar Typhimurium. Jpn J Infect Dis 56:151–155. 14583637

[80] West DM, Sprigings KA, Cassar C, Wakeley PR, Sawyer J, Davies RH. 2007. Rapid detection of Escherichia coli virulence factor genes using multiplex real-time TaqMan PCR assays. Vet Microbiol 122:323–331. doi:10.1016/j.vetmic.2007.01.026. 17336470

[81] Tsen HY, Chi WR, Lin CK. 1996. Use of novel polymerase chain reaction primers for the specific detection of heat-labile toxin I, heat-stable toxin I and II enterotoxigenic Escherichia coli in milk. J Food Prot 59:795–802. doi:10.4315/0362-028X-59.8.795. 31159120

[82] Kong RYC, So CL, Law WF, Wu RSS. 1999. A sensitive and versatile multiplex PCR system for the rapid detection of enterotoxigenic (ETEC), enterohaemorrhagic (EHEC) and enteropathogenic (EPEC) strains of Escherichia coli. Mar Pollut Bull 38:1207–1215. doi:10.1016/S0025-326X(99)00164-2.

[83] Ngeleka M, Pritchard J, Appleyard G, Middleton DM, Fairbrother JM. 2003. Isolation and association of Escherichia coli AIDA-I/STb, rather than EAST1 pathotype, with diarrhea in piglets and antibiotic sensitivity of isolates. J Vet Diagn Invest 15:242–252. doi:10.1177/104063870301500305. 12735346

[84] Sharma VK, Dean-Nystrom EA. 2003. Detection of enterohemorrhagic Escherichia coli O157:H7 by using a multiplex real-time PCR assay for genes encoding intimin and Shiga toxins. Vet Microbiol 93:247–260. doi:10.1016/s0378-1135(03)00039-7. 12695048

[85] Paton AW, Paton JC. 1998. Detection and characterization of Shiga toxigenic Escherichia coli by using multiplex PCR assays for stx1, stx2, eaeA, enterohemorrhagic E. coli hlyA, rfbO111, and rfbO157. J Clin Microbiol 36:598–602. doi:10.1128/JCM.36.2.598-602.1998. 9466788 PMC104589

