# Supplementary material for: Calf Diarrhea Caused by Prolonged Expansion of Autochthonous Gut Enterobacteriaceae and Their Lytic Bacteriophages
Source: mSystems. 2021 Mar 2;6(2):e00816-20. doi: 10.1128/mSystems.00816-20 (PMC8546982; doi:10.1128/mSystems.00816-20)
Supplement: TABLE S1 [file msystems.00816-20-st001.pdf]

TABLE S1 Calf age and sex, and moisture content of the collected samples.

Sample ID	Status	n	Sex	Age (day after birth)	Moisture content (%)	Analysis type
N1	Normal	1	Female	27	53.68	RNA-Seq, 16S rRNA sequencing, and diagnostic PCR
N2	Normal	1	Female	48	65.60	RNA-Seq, 16S rRNA sequencing, and diagnostic PCR
N3	Normal	1	Male	24	73.72	RNA-Seq, 16S rRNA sequencing, and diagnostic PCR
N4	Normal	1	Male	24	65.53	RNA-Seq, 16S rRNA sequencing, and diagnostic PCR
N5	Normal	1	Female	35	67.47	RNA-Seq, 16S rRNA sequencing, and diagnostic PCR
N6	Normal	1	Female	22	56.42	RNA-Seq, 16S rRNA sequencing, and diagnostic PCR
N7	Normal	1	Male	42	50.21	RNA-Seq, 16S rRNA sequencing, and diagnostic PCR
N8	Normal	1	Female	28	52.78	RNA-Seq, 16S rRNA sequencing, and diagnostic PCR
N9	Normal	1	Male	33	77.58	RNA-Seq, 16S rRNA sequencing, and diagnostic PCR
N10	Normal	1	Female	15	51.19	16S rRNA sequencing and diagnostic PCR
N11	Normal	1	Female	15	60.43	16S rRNA sequencing and diagnostic PCR
N12	Normal	1	Female	19	60.01	16S rRNA sequencing and diagnostic PCR
N13	Normal	1	Male	10	56.49	16S rRNA sequencing and diagnostic PCR
N14	Normal	1	Male	11	53.12	16S rRNA sequencing and diagnostic PCR
N15	Normal	1	Female	21	53.90	16S rRNA sequencing and diagnostic PCR
N16	Normal	1	Female	16	64.08	16S rRNA sequencing and diagnostic PCR
N17	Normal	1	Male	17	51.13	16S rRNA sequencing and diagnostic PCR
N18	Normal	1	Female	15	54.98	16S rRNA sequencing and diagnostic PCR
N19	Normal	1	Male	15	53.74	16S rRNA sequencing and diagnostic PCR
N20	Normal	1	Male	11	53.22	16S rRNA sequencing and diagnostic PCR
N21	Normal	1	Female	22	52.93	16S rRNA sequencing and diagnostic PCR
N22	Normal	1	Female	20	51.16	16S rRNA sequencing and diagnostic PCR
N23	Normal	1	Female	19	58.55	16S rRNA sequencing and diagnostic PCR
N24	Normal	1	Male	18	57.32	16S rRNA sequencing and diagnostic PCR
N25	Normal	1	Male	18	54.09	16S rRNA sequencing and diagnostic PCR
N26	Normal	1	Male	11	53.87	16S rRNA sequencing and diagnostic PCR
N27	Normal	1	Male	29	59.82	16S rRNA sequencing and diagnostic PCR
N28	Normal	1	Female	22	54.52	16S rRNA sequencing and diagnostic PCR
N29	Normal	1	Male	24	52.69	16S rRNA sequencing and diagnostic PCR
N30	Normal	1	Female	28	53.19	16S rRNA sequencing and diagnostic PCR
N31	Normal	1	Male	13	63.51	16S rRNA sequencing and diagnostic PCR
N32	Normal	1	Male	14	57.48	16S rRNA sequencing and diagnostic PCR
N33	Normal	1	Male	20	58.22	16S rRNA sequencing and diagnostic PCR
N34	Normal	1	Male	17	53.54	16S rRNA sequencing and diagnostic PCR
N35	Normal	1	Male	27	59.25	16S rRNA sequencing and diagnostic PCR
N36	Normal	1	Female	15	58.48	16S rRNA sequencing and diagnostic PCR
N37	Normal	1	Female	26	54.22	16S rRNA sequencing and diagnostic PCR
N38	Normal	1	Male	13	60.29	16S rRNA sequencing and diagnostic PCR
N39	Normal	1	Female	15	50.38	16S rRNA sequencing and diagnostic PCR
N40	Normal	1	Male	16	52.62	16S rRNA sequencing and diagnostic PCR
N41	Normal	1	Male	13	51.87	16S rRNA sequencing and diagnostic PCR
N42	Normal	1	Female	15	58.64	16S rRNA sequencing and diagnostic PCR
N43	Normal	1	Female	11	56.92	16S rRNA sequencing and diagnostic PCR
N44	Normal	1	Male	21	54.00	16S rRNA sequencing and diagnostic PCR
N45	Normal	1	Female	25	54.83	16S rRNA sequencing and diagnostic PCR

N46	Normal	1	Male	18	56.99	16S rRNA sequencing and diagnostic PCR
N47	Normal	1	Male	22	53.27	16S rRNA sequencing and diagnostic PCR
N48	Normal	1	Male	20	54.54	16S rRNA sequencing and diagnostic PCR
N49	Normal	1	Female	24	54.68	16S rRNA sequencing and diagnostic PCR
N50	Normal	1	Male	34	54.29	16S rRNA sequencing and diagnostic PCR
N51	Normal	1	Male	31	60.38	16S rRNA sequencing and diagnostic PCR
N52	Normal	1	Female	36	59.27	16S rRNA sequencing and diagnostic PCR
N53	Normal	1	Male	48	53.12	16S rRNA sequencing and diagnostic PCR
D1	Diarrhea	1	Male	34	90.63	RNA-Seq, 16S rRNA sequencing, and diagnostic PCR
D2	Diarrhea	1	Male	34	89.19	RNA-Seq, 16S rRNA sequencing, and diagnostic PCR
D3	Diarrhea	1	Female	24	93.18	RNA-Seq, 16S rRNA sequencing, and diagnostic PCR
D4	Diarrhea	1	Male	34	92.85	RNA-Seq, 16S rRNA sequencing, and diagnostic PCR
D5	Diarrhea	1	Male	29	95.85	RNA-Seq, 16S rRNA sequencing, and diagnostic PCR
D6	Diarrhea	1	Male	31	89.65	RNA-Seq, 16S rRNA sequencing, and diagnostic PCR
D7	Diarrhea	1	Female	22	90.33	RNA-Seq, 16S rRNA sequencing, and diagnostic PCR
D8	Diarrhea	1	Female	33	95.76	RNA-Seq, 16S rRNA sequencing, and diagnostic PCR
D9	Diarrhea	1	Male	22	94.80	RNA-Seq, 16S rRNA sequencing, and diagnostic PCR
D10	Diarrhea	1	Male	16	92.38	16S rRNA sequencing and diagnostic PCR
D11	Diarrhea	1	Female	16	88.12	16S rRNA sequencing and diagnostic PCR
D12	Diarrhea	1	Male	17	89.39	16S rRNA sequencing and diagnostic PCR
D13	Diarrhea	1	Female	15	91.55	16S rRNA sequencing and diagnostic PCR
D14	Diarrhea	1	Male	11	92.78	16S rRNA sequencing and diagnostic PCR
D15	Diarrhea	1	Male	21	92.53	16S rRNA sequencing and diagnostic PCR
D16	Diarrhea	1	Female	25	92.85	16S rRNA sequencing and diagnostic PCR
D17	Diarrhea	1	Male	28	90.11	16S rRNA sequencing and diagnostic PCR
D18	Diarrhea	1	Female	24	91.67	16S rRNA sequencing and diagnostic PCR
D19	Diarrhea	1	Male	20	91.03	16S rRNA sequencing and diagnostic PCR
D20	Diarrhea	1	Male	9	94.12	16S rRNA sequencing and diagnostic PCR
D21	Diarrhea	1	Male	16	93.79	16S rRNA sequencing and diagnostic PCR
D22	Diarrhea	1	Female	15	92.88	16S rRNA sequencing and diagnostic PCR
D23	Diarrhea	1	Female	23	92.64	16S rRNA sequencing and diagnostic PCR
D24	Diarrhea	1	Male	19	91.10	16S rRNA sequencing and diagnostic PCR
D25	Diarrhea	1	Male	21	85.17	16S rRNA sequencing and diagnostic PCR
D26	Diarrhea	1	Female	27	88.14	16S rRNA sequencing and diagnostic PCR
D27	Diarrhea	1	Female	17	91.55	16S rRNA sequencing and diagnostic PCR
D28	Diarrhea	1	Female	10	89.30	16S rRNA sequencing and diagnostic PCR
D29	Diarrhea	1	Male	21	87.11	16S rRNA sequencing and diagnostic PCR
D30	Diarrhea	1	Male	28	94.16	16S rRNA sequencing and diagnostic PCR
D31	Diarrhea	1	Female	22	91.01	16S rRNA sequencing and diagnostic PCR
D32	Diarrhea	1	Female	29	92.55	16S rRNA sequencing and diagnostic PCR
D33	Diarrhea	1	Male	21	92.93	16S rRNA sequencing and diagnostic PCR
D34	Diarrhea	1	Female	24	89.75	16S rRNA sequencing and diagnostic PCR
D35	Diarrhea	1	Male	31	88.96	16S rRNA sequencing and diagnostic PCR
D36	Diarrhea	1	Male	25	91.74	16S rRNA sequencing and diagnostic PCR
D37	Diarrhea	1	Male	25	92.66	16S rRNA sequencing and diagnostic PCR
D38	Diarrhea	1	Female	25	92.19	16S rRNA sequencing and diagnostic PCR
D39	Diarrhea	1	Male	25	90.10	16S rRNA sequencing and diagnostic PCR
D40	Diarrhea	1	Male	13	91.37	16S rRNA sequencing and diagnostic PCR

D41	Diarrhea	1	Female	19	94.88	16S rRNA sequencing and diagnostic PCR
D42	Diarrhea	1	Female	29	93.65	16S rRNA sequencing and diagnostic PCR
D43	Diarrhea	1	Female	14	91.12	16S rRNA sequencing and diagnostic PCR
D44	Diarrhea	1	Male	13	92.33	16S rRNA sequencing and diagnostic PCR
D45	Diarrhea	1	Female	13	90.08	16S rRNA sequencing and diagnostic PCR
D46	Diarrhea	1	Male	18	89.36	16S rRNA sequencing and diagnostic PCR
D47	Diarrhea	1	Male	19	88.16	16S rRNA sequencing and diagnostic PCR
D48	Diarrhea	1	Female	22	84.39	16S rRNA sequencing and diagnostic PCR
D49	Diarrhea	1	Female	22	91.28	16S rRNA sequencing and diagnostic PCR
D50	Diarrhea	1	Male	21	91.12	16S rRNA sequencing and diagnostic PCR
D51	Diarrhea	1	Female	29	91.71	16S rRNA sequencing and diagnostic PCR
D52	Diarrhea	1	Male	31	91.44	16S rRNA sequencing and diagnostic PCR
D53	Diarrhea	1	Female	24	90.48	16S rRNA sequencing and diagnostic PCR
p-value	-	-	0.4985*	0.1460*	0.0095§	-
#ND1	N-D repeated	5	Male	14-29	N/A	16S rRNA sequencing
#ND2	N-D repeated	5	Female	27-42	N/A	16S rRNA sequencing
#ND3	N-D repeated	5	Male	15-30	N/A	16S rRNA sequencing
#ND4	N-D repeated	4	Female	19-26	N/A	16S rRNA sequencing
#ND5	N-D repeated	4	Male	14-21	N/A	16S rRNA sequencing

Abbreviations: N, normal; D, diarrhea.

*The data were analyzed using the non-parametric Mann-Whitney *U* test (one-tailed).

§The data were analyzed using the Chi-square test.

#Animals with repeated normal diarrhea.
