# Supplementary figures and images for: Calf Diarrhea Caused by Prolonged Expansion of Autochthonous Gut Enterobacteriaceae and Their Lytic Bacteriophages
Source: mSystems. 2021 Mar 2;6(2):e00816-20. doi: 10.1128/mSystems.00816-20 (PMC8546982; doi:10.1128/mSystems.00816-20)

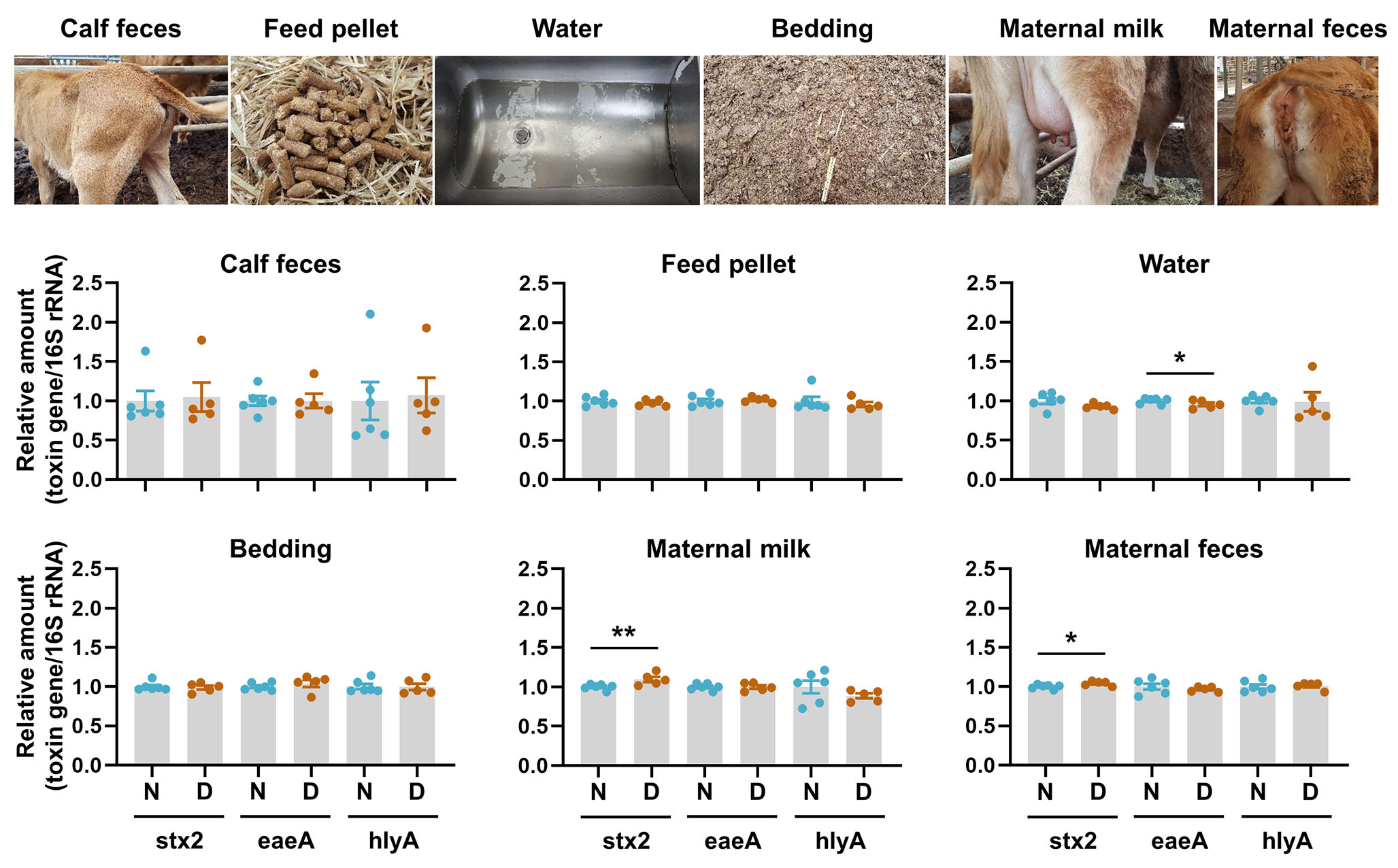

Supplement: FIG S1 [file msystems.00816-20-sf001.jpg]

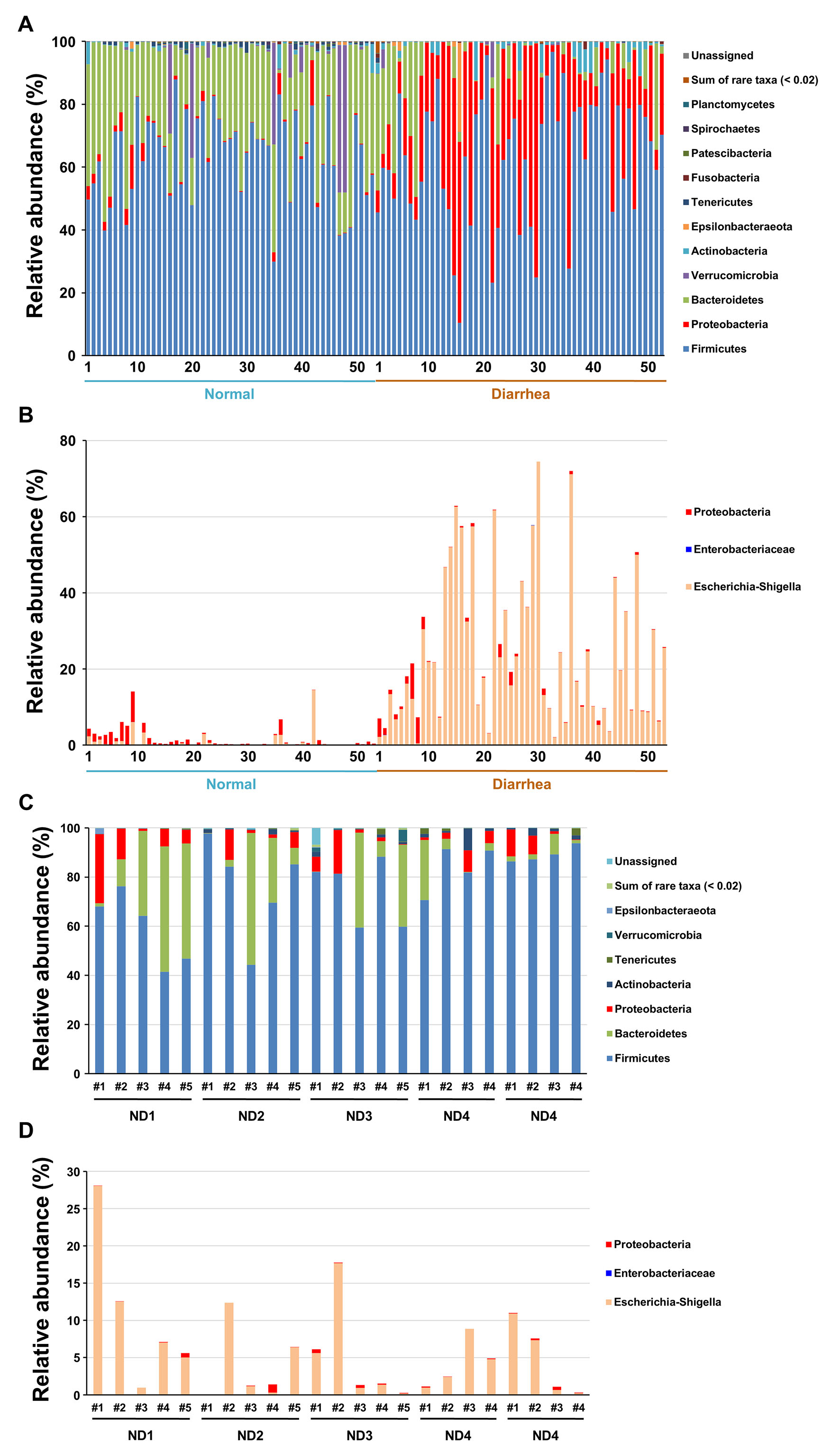

Supplement: FIG S2 [file msystems.00816-20-sf002.jpg]

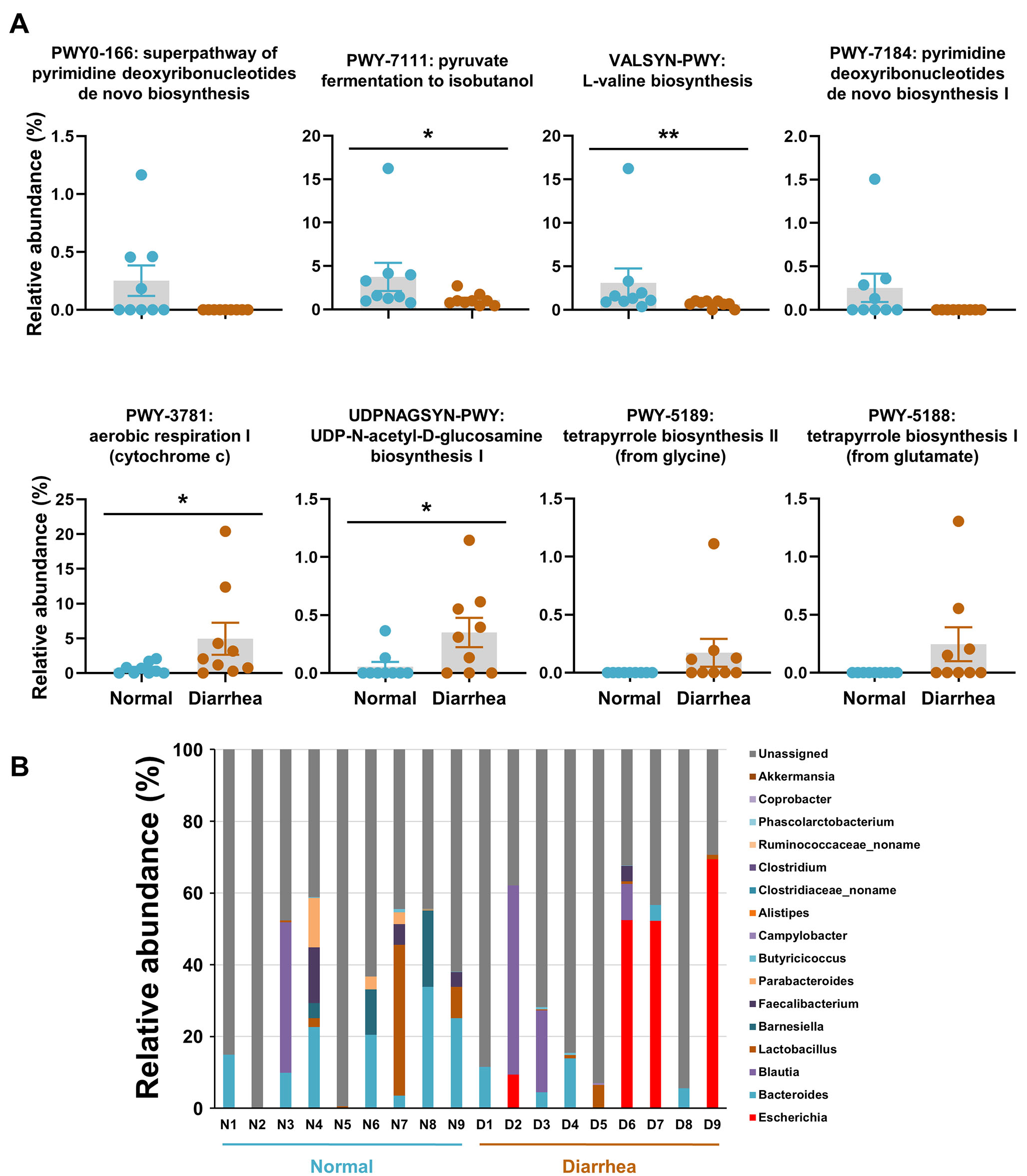

Supplement: FIG S3 [file msystems.00816-20-sf003.jpg]

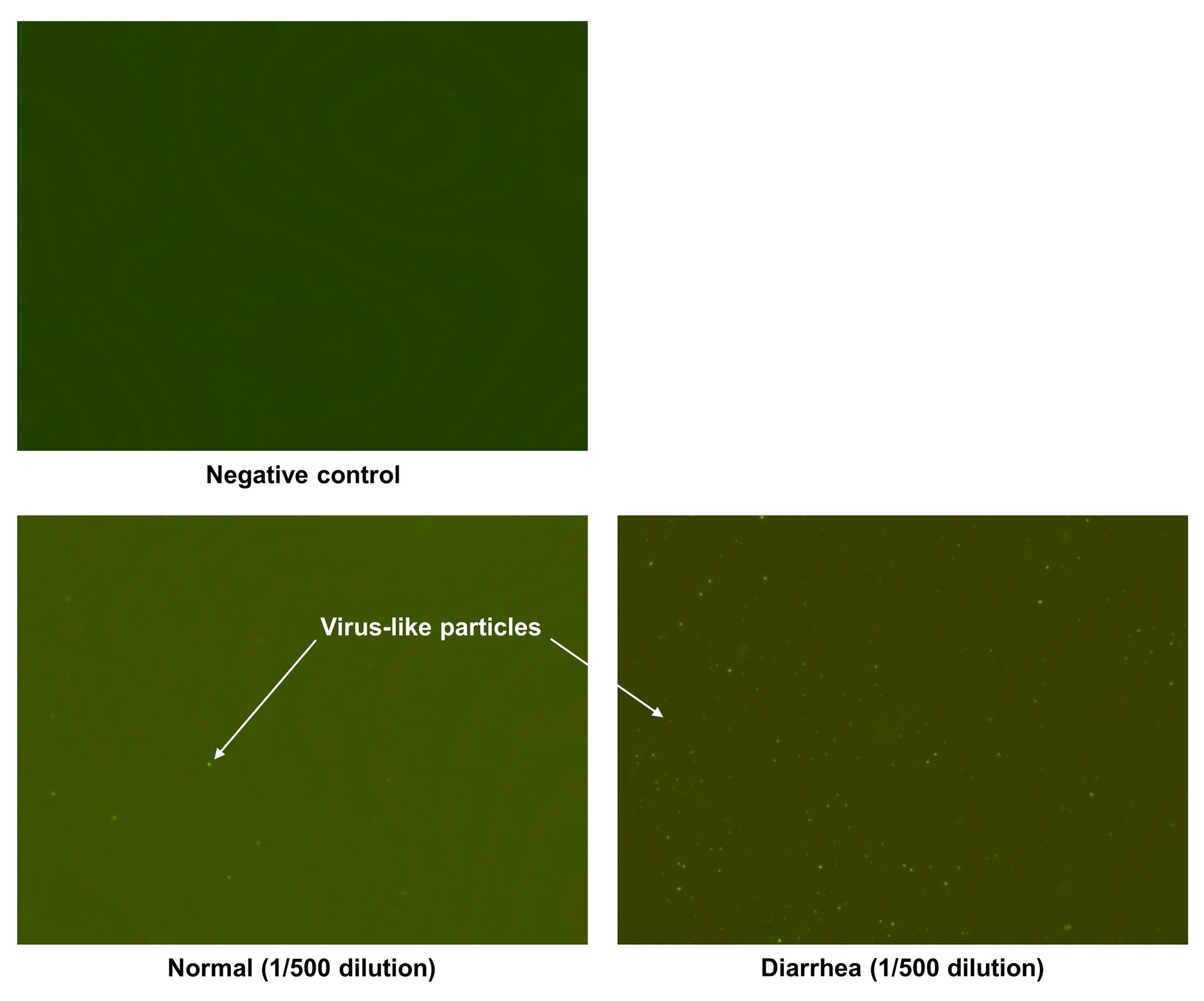

Supplement: FIG S4 [file msystems.00816-20-sf004.jpg]

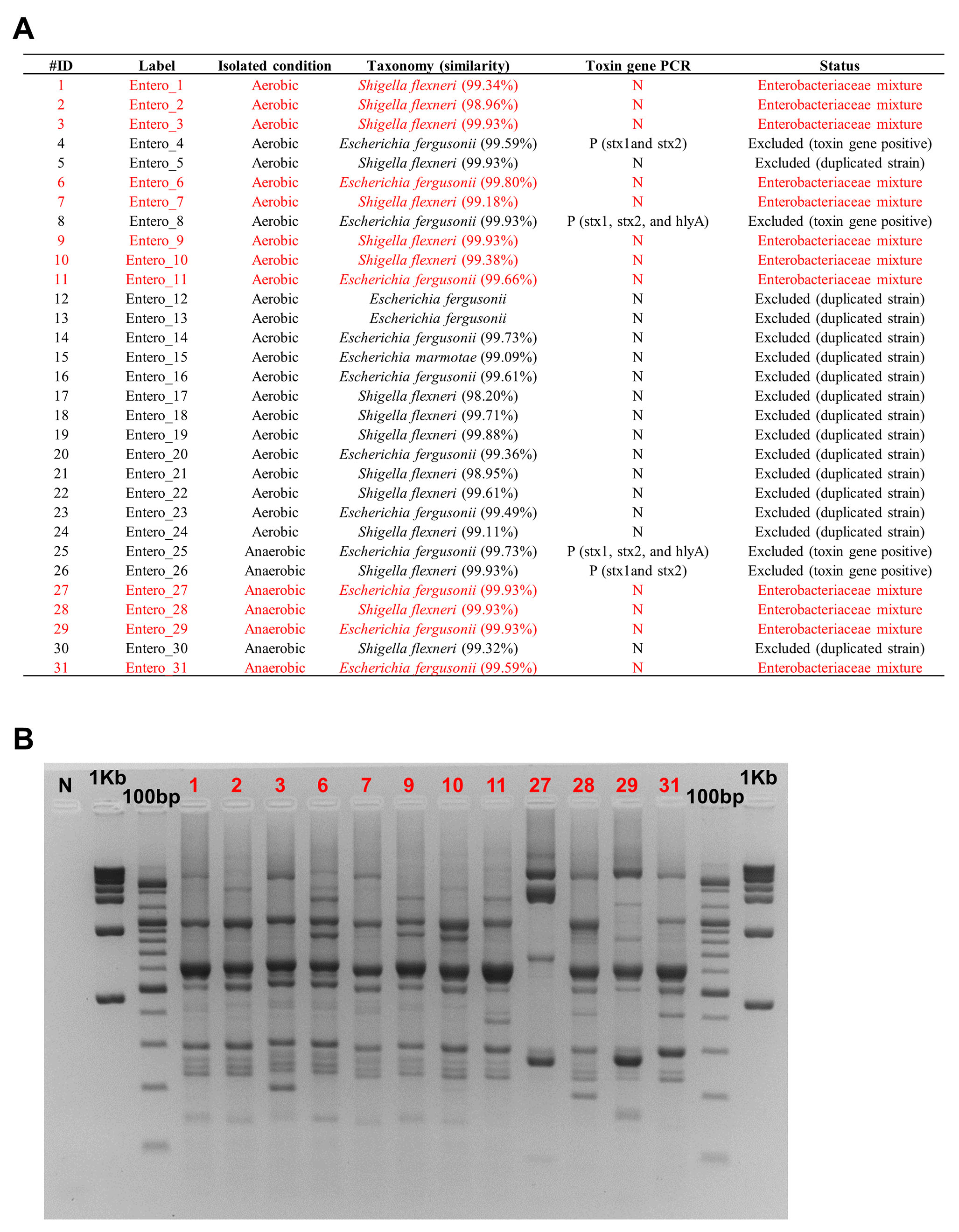

Supplement: FIG S5 [file msystems.00816-20-sf005.jpg]

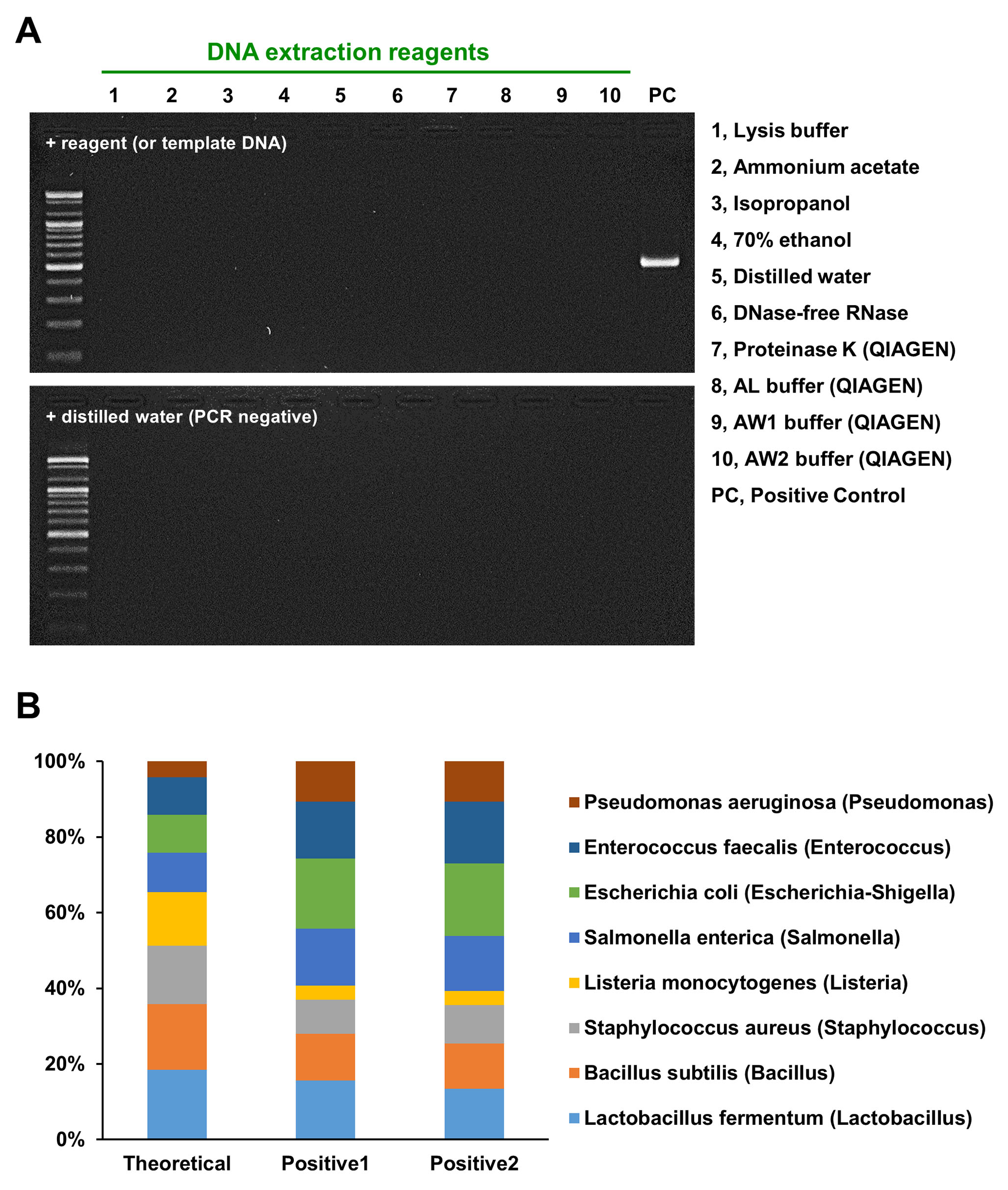

Supplement: FIG S6 [file msystems.00816-20-sf006.jpg]
