# Supplementary material for: Calf Diarrhea Caused by Prolonged Expansion of Autochthonous Gut Enterobacteriaceae and Their Lytic Bacteriophages
Source: mSystems. 2021 Mar 2;6(2):e00816-20. doi: 10.1128/mSystems.00816-20 (PMC8546982; doi:10.1128/mSystems.00816-20)
Supplement: TABLE S2 [file msystems.00816-20-st002.pdf]

TABLE S2 Results of the diagnostic multiplex PCR analysis of samples from the calf environment.

		Target gene	**Normal**						**Diarrhea**				
			N1	N2	N3	N4	N5	N6	D1	D2	D3	D4	D5
**Calf husbandry samples**	Calf feces	*stx2*	+									+	
		eaeA	+								+	+	
		hlyA	+	+	+							+	+
	Feed pellet	*stx2*											
		eaeA											
		hlyA											
	Water	*stx2*											
		eaeA											
		hlyA											
	Bedding	*stx2*											
		eaeA						+					
		hlyA											
**Maternal samples**	Milk	*stx2*											
		eaeA											
		hlyA											
	Feces	*stx2*											
		eaeA											
		hlyA											

Abbreviations: stx2, Shiga toxin type 2; eaeA, enterohemorrhagic E. coli O157:H7-specific intimin; hlyA, plasmid-encoded enterohemolysin.
