# Supplementary material for: Calf Diarrhea Caused by Prolonged Expansion of Autochthonous Gut Enterobacteriaceae and Their Lytic Bacteriophages
Source: mSystems. 2021 Mar 2;6(2):e00816-20. doi: 10.1128/mSystems.00816-20 (PMC8546982; doi:10.1128/mSystems.00816-20)
Supplement: TABLE S3 [file msystems.00816-20-st003.pdf]

TABLE S3 Taxonomic annotation data for the negative control.

OTU ID	The number of reads		Taxonomy (QIIME)
	*Negative1	Negative2	
109057	9	0	p_Proteobacteria; c_Deltaproteobacteria; o_Myxococcales; f_0319-6G20; g_; s_
denovo_70	0	2	p_Proteobacteria; c_Gammaproteobacteria; o_Pseudomonadales; f_Moraxellaceae; g_Acinetobacter; s_
denovo_71	0	1	p_Proteobacteria; c_Gammaproteobacteria; o_Pseudomonadales; f_Moraxellaceae; g_Acinetobacter; s_
1033426	19	0	p_Actinobacteria; c_Actinobacteria; o_Actinomycetales; f_Nocardioidaceae; g_Nocardioides; s_
572237	13	0	p_Fusobacteria; c_Fusobacteriia; o_Fusobacteriales; f_Fusobacteriaceae; g_Fusobacterium; s_
denovo_91	0	1	p_Proteobacteria; c_Gammaproteobacteria; o_Pseudomonadales; f_Moraxellaceae; g_Acinetobacter; s_
denovo_26	0	6	p_Proteobacteria; c_Gammaproteobacteria; o_Pseudomonadales; f_Moraxellaceae; g_Acinetobacter; s_
denovo_20	0	1	p_Proteobacteria; c_Gammaproteobacteria; o_Pseudomonadales; f_Moraxellaceae; g_Acinetobacter; s_
1144747	23	0	p_Bacteroidetes; c_Flavobacteriia; o_Flavobacteriales; f_[Weeksellaceae]; g_Chryseobacterium; s_
992354	20	19	p_Actinobacteria; c_Actinobacteria; o_Actinomycetales; f_Propionibacteriaceae; g_Propionibacterium; s_acnes
4458848	6	0	p_Proteobacteria; c_Deltaproteobacteria; o_Myxococcales; f_0319-6G20; g_; s_
50109	0	15	p_Verrucomicrobia; c_[Pedosphaerae]; o_[Pedosphaerales]; f_Ellin517; g_; s_
3574065	0	1	p_Cyanobacteria; c_Chloroplast; o_Streptophyta; f_; g_; s_
515265	0	1	p_Proteobacteria; c_Gammaproteobacteria; o_Pseudomonadales; f_Moraxellaceae; g_Acinetobacter; s_
denovo_139	0	1	p_Proteobacteria; c_Gammaproteobacteria; o_Pseudomonadales; f_Moraxellaceae; g_Acinetobacter; s_
4458619	0	2	p_Actinobacteria; c_Actinobacteria; o_Actinomycetales; f_Dermacoccaceae; g_Dermacoccus; s_
992510	0	2	p_Proteobacteria; c_Alphaproteobacteria; o_Sphingomonadales; f_Sphingomonadaceae; g_; s_
588097	4	0	p_Proteobacteria; c_Gammaproteobacteria; o_Xanthomonadales; f_Xanthomonadaceae; g_Stenotrophomonas; s_
646549	0	15	p_Proteobacteria; c_Gammaproteobacteria; o_Pseudomonadales; f_Pseudomonadaceae; g_Pseudomonas; s_
1108960	0	22	p_Proteobacteria; c_Alphaproteobacteria; o_Sphingomonadales; f_Sphingomonadaceae; g_Sphingomonas; s_
1131954	15	0	p_Cyanobacteria; c_Synechococcophycideae; o_Pseudanabaenales; f_Pseudanabaenaceae; g_; s_
3774028	21	0	p_Proteobacteria; c_Alphaproteobacteria; o_Caulobacterales; f_Caulobacteraceae; g_; s_
113674	34	0	p_Bacteroidetes; c_Sphingobacteriia; o_Sphingobacteriales; f_; g_; s_
1091060	0	16	p_Proteobacteria; c_Alphaproteobacteria; o_Sphingomonadales; f_Sphingomonadaceae; g_Sphingomonas; s_
2307137	0	2	p_Cyanobacteria; c_Chloroplast; o_Streptophyta; f_; g_; s_
denovo_49	0	15	p_Proteobacteria; c_Alphaproteobacteria; o_; f_; g_; s_
denovo_47	0	1	p_Proteobacteria; c_Gammaproteobacteria; o_Pseudomonadales; f_Moraxellaceae; g_Acinetobacter; s_
591966	31	2	p_Cyanobacteria; c_4C0d-2; o_MLE1-12; f_; g_; s_
1106466	44	0	p_Bacteroidetes; c_Cytophagia; o_Cytophagales; f_Flammeovirgaceae; g_; s_
498063	0	10	p_Proteobacteria; c_Gammaproteobacteria; o_Xanthomonadales; f_Xanthomonadaceae; g_Luteimonas; s_
287179	0	4	p_Proteobacteria; c_Betaproteobacteria; o_Burkholderiales; f_Comamonadaceae; g_; s_
68458	0	3	p_Proteobacteria; c_Alphaproteobacteria; o_Rhizobiales; f_Methylobacteriaceae; g_Methylobacterium; s_
denovo_65	0	1	p_Proteobacteria; c_Betaproteobacteria; o_Burkholderiales; f_Comamonadaceae; g_Delftia; s_
denovo_64	0	1	p_Proteobacteria; c_Gammaproteobacteria; o_Pseudomonadales; f_Moraxellaceae; g_Acinetobacter; s_
denovo_89	0	7	p_Proteobacteria; c_Betaproteobacteria; o_Burkholderiales; f_Comamonadaceae; g_Acidovorax
denovo_88	0	1	p_Proteobacteria; c_Gammaproteobacteria; o_Pseudomonadales; f_Moraxellaceae; g_Acinetobacter; s_
1640195	24	0	p_Proteobacteria; c_Alphaproteobacteria; o_Rhodospirillales; f_Rhodospirillaceae; g_; s_
denovo_12	0	2	p_Proteobacteria; c_Gammaproteobacteria; o_Pseudomonadales; f_Moraxellaceae; g_Acinetobacter; s_
627435	5	0	p_Proteobacteria; c_Deltaproteobacteria; o_Myxococcales; f_0319-6G20; g_; s_

822806	5	0	p_Chloroflexi; c_Anaerolineae; o_SBR1031; f_A4b; g_; s_
590586	0	20	p_Proteobacteria; c_Alphaproteobacteria; o_Rhodobacterales; f_Rhodobacteraceae; g_Paracoccus; s_
226053	0	16	p_Cyanobacteria; c_Chloroplast; o_Streptophyta; f_; g_; s_
208315	0	4	p_Cyanobacteria; c_Oscillatoriophycideae; o_Oscillatoriales; f_Phormidiaceae; g_Phormidium; s_
405425	0	1	p_Proteobacteria; c_Gammaproteobacteria; o_Pseudomonadales; f_Moraxellaceae; g_Acinetobacter; s_
888396	0	7	p_Proteobacteria; c_Betaproteobacteria; o_Burkholderiales; f_Comamonadaceae; g_; s_
720353	0	9	p_Proteobacteria; c_Betaproteobacteria; o_Burkholderiales; f_Comamonadaceae; g_Delftia; s_
1021984	0	4	p_Proteobacteria; c_Deltaproteobacteria; o_Myxococcales; f_; g_; s_
150994	13	0	p_Bacteroidetes; c_Sphingobacteriia; o_Sphingobacteriales; f_; g_; s_
278371	7	0	p_Cyanobacteria; c_Oscillatoriophycideae; o_Oscillatoriales; f_Phormidiaceae; g_Phormidium; s_
1040713	0	6	p_Actinobacteria; c_Actinobacteria; o_Actinomycetales; f_Corynebacteriaceae; g_Corynebacterium; s_
denovo_123	0	1	p_Proteobacteria; c_Gammaproteobacteria; o_Pseudomonadales; f_Moraxellaceae; g_Acinetobacter; s_
denovo_124	0	1	p_Proteobacteria; c_Gammaproteobacteria; o_Pseudomonadales; f_Moraxellaceae; g_Acinetobacter; s_
denovo_32	0	2	p_Proteobacteria; c_Gammaproteobacteria; o_Pseudomonadales; f_Moraxellaceae; g_Acinetobacter; s_
denovo_33	0	1	p_Proteobacteria; c_Gammaproteobacteria; o_Pseudomonadales; f_Moraxellaceae; g_Acinetobacter; s_
1076316	0	4	p_Firmicutes; c_Bacilli; o_Bacillales; f_Staphylococcaceae; g_Staphylococcus; s_
1060029	0	9	p_Actinobacteria; c_Actinobacteria; o_Actinomycetales; f_Corynebacteriaceae; g_Corynebacterium; s_
582973	0	12	p_Proteobacteria; c_Alphaproteobacteria; o_Sphingomonadales; f_Erythrobacteraceae; g_; s_
1008747	14	1	p_Proteobacteria; c_Betaproteobacteria; o_Burkholderiales; f_Oxalobacteraceae; g_; s_
denovo_100	0	39	p_Proteobacteria; c_Gammaproteobacteria; o_Pseudomonadales; f_Moraxellaceae; g_Acinetobacter; s_
denovo_105	0	2	p_Proteobacteria; c_Gammaproteobacteria; o_Pseudomonadales; f_Moraxellaceae; g_Acinetobacter; s_
545997	0	11	p_Proteobacteria; c_Betaproteobacteria; o_Burkholderiales; f_Comamonadaceae; g_; s_
denovo_50	0	7	p_Proteobacteria; c_Gammaproteobacteria; o_Pseudomonadales; f_Moraxellaceae; g_Acinetobacter; s_
593096	0	14	p_Proteobacteria; c_Alphaproteobacteria; o_Rhodospirillales; f_Acetobacteraceae; g_; s_
607212	2	0	p_Proteobacteria; c_Alphaproteobacteria; o_Sphingomonadales; f_Sphingomonadaceae; g_Sphingomonas; s_
900973	0	4	p_Proteobacteria; c_Betaproteobacteria; o_Neisseriales; f_Neisseriaceae; g_; s_
808758	20	0	p_Proteobacteria; c_Alphaproteobacteria; o_Sphingomonadales; f_Sphingomonadaceae; g_Novosphingobium; s_
Total	329	329	

* "Blank" negative DNA extraction/PCR controls (i.e., PCR products of template acquired from a sham extraction to which no rectal luminal sample is added).
